# High-field multinuclear MAS NMR and synchrotron XANES reveal the influence of strontium salt chemistry on geopolymer nanostructure

**DOI:** 10.1039/d6dt00775a

**Published:** 2026-06-17

**Authors:** Kyle T. O'Donoghue, Daniel A. Geddes, Tom J. Wilkinson, Martin C. Stennett, Byoungkwan Kim, Dinu Iuga, Martin Hayes, Brant Walkley

**Affiliations:** a School of Chemical, Materials and Biological Engineering, The University of Sheffield Sheffield UK b.walkley@sheffield.ac.uk; b Department of Physics, University of Warwick Coventry UK; c United Kingdom National Nuclear Laboratory Warrington UK

## Abstract

This study investigates the influence of strontium (Sr) salt chemistry (Sr(OH)_2_·8H_2_O, SrCO_3_, Sr(NO_3_)_2_, and SrSO_4_) on the nanostructural evolution of potassium silicate-activated geopolymers. High-field multinuclear (^27^Al, ^29^Si, ^39^K, and ^87^Sr) MAS NMR, synchrotron XANES, EPMA, XRD, and FTIR showed that while the primary binding phase in all samples is a disordered, highly cross-linked K–A–S–H gel, the Sr immobilisation mechanism is governed by salt solubility. Soluble nitrate and hydroxide salts release Sr^2+^ ions that are chemically incorporated into the K–A–S–H gel framework in brewsterite-type pseudo-zeolitic environments. In contrast, insoluble carbonate and sulfate salts act primarily as physical fillers, and are encapsulated as discrete particles within the geopolymer matrix, though sulfate additionally reacts to form secondary crystalline kalistrontite (K_2_Sr(SO_4_)_2_). Sr^2+^ adsorption on metakaolin surfaces is found to inhibit early-stage Al dissolution, resulting in a Si-rich K–A–S–H gel that transitions to an Al-rich K–A–S–H gel over 28 days. These results provide new insight into the mechanisms of immobilisation of Sr in geopolymers, and highlight their potential as wasteforms for long-term management of ^90^Sr radioactive waste.

## Abstract 2

This study investigates the influence of strontium (Sr) salt chemistry (Sr(OH)_2_·8H_2_O, SrCO_3_, Sr(NO_3_)_2_, and SrSO_4_) on the nanostructural evolution of potassium silicate-activated geopolymers.

## Graphical abstract

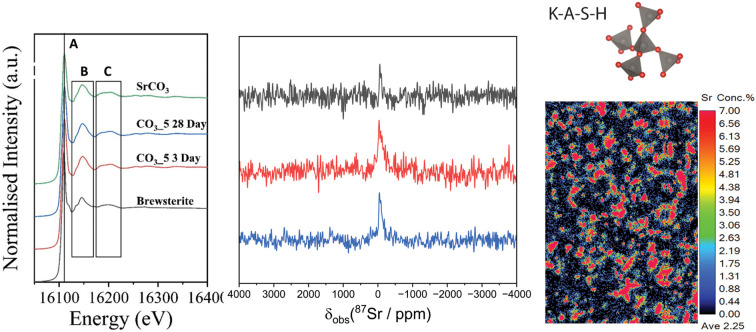

## 1 Introduction

Nuclear power generation produces a wide range of radioactive waste streams that require appropriate conditioning prior to secure disposal. Among these wastes is strontium-90 (^90^Sr), a fission product commonly present in reactor cooling water and in complex legacy waste inventories at facilities such as Sellafield and Fukushima Daiichi. Ensuring the safe, long-term disposal of ^90^Sr is a global priority as efforts to develop deep geological disposal facilities (GDFs) and other disposal scenarios progress.^1^ Developing a suitable wasteform capable of providing a robust engineered barrier for ^90^Sr is key to achieving this.

Cementation offers a low-cost, low-temperature method for conditioning wastes containing ^90^Sr.^2–4^ Because ^90^Sr in aqueous waste streams is frequently extracted using granular ion-exchange materials, cement-based encapsulation is particularly compatible with this waste type. Portland cement (PC) has traditionally been used for this purpose and is known to bind Sr through chemisorption onto the calcium silicate hydrate (C–S–H) gel phases. The substitution of Ca by ^90^Sr within PC hydrate phases, especially C–S–H, has also been reported.^2,5^

Much of the ^90^Sr retention in PC arises from reversible ion-exchange interactions with hydrated or partially hydrated phases in the hardened cement matrix.^5^ This limits the extent of true chemical immobilisation, leaving a significant proportion of Sr physically trapped rather than chemically incorporated. Additionally, the high water content of hydrated PC raises concerns regarding radiolytic stability.^6^ The substantial CO_2_ emissions associated with PC production (accounting for roughly 8% of global anthropogenic CO_2_ output) represent another growing concern within the nuclear sector in recent years, particularly regarding long-term sustainability.^7,8^ These issues highlight the need for alternative cementitious systems that can provide sustainable, high-performance immobilisation of ^90^Sr-bearing wastes.

Geopolymers are promising alternatives to PC-based encapsulants. Their disordered, highly cross-linked aluminosilicate gel network contains cation-binding sites capable of incorporating species such as Sr^2+^ through ion-exchange processes.^9,10^ Geopolymers also exhibit excellent radiolytic stability^11^ and possess favourable chemical, mechanical, and thermal characteristics, driving significant interest in their use for radioactive waste immobilisation.

Sodium and potassium are the alkali metals most commonly used in geopolymer synthesis, producing aluminosilicate hydrate gel networks collectively referred to as (N,K)–A–S–H. The nanostructure of these gels is often described as pseudo-zeolitic: while short-range ordering resembles that of zeolites (on the scale of a few Å),^12,13^ the long-range structure is highly disordered and X-ray amorphous. As in zeolites, Si and Al occupy tetrahedral coordination environments, with Si typically present in Q^4^(*m*Al) environments and Al most commonly present in Q^4^(4Si) coordination. The negative charge introduced by each AlO_4_ tetrahedron is balanced by alkali cations.^14^

Although previous studies have examined interactions between sodium-activated geopolymers and Sr-bearing wastes, potassium-activated systems have demonstrated superior flow behaviour and rheological properties compared with sodium-based formulations.^15–17^ Previous work on potassium-based geopolymers incorporating various strontium salts showed that the anion associated with the strontium salts (Sr(OH)_2_·8H_2_O, SrCO_3_, Sr(NO_3_)_2_, and SrSO_4_) strongly affected geopolymer reaction mechanisms and kinetics in the fresh geopolymer paste,^18^ and was controlled in particular by salt solubility. However, the radionuclide incorporation processes which control long-term performance of these materials remain unclear. In particular, there has been little investigation of the influence of the anion associated with salts of strontium on the Sr incorporation mechanism and nanostructural evolution in potassium silicate-based geopolymers in the hardened wasteform.

Solid-state magic angle spinning nuclear magnetic resonance (MAS NMR) spectroscopy investigations of ^27^Al, ^29^Si, in geopolymers^19–22^ and synthetic N–A–S–H^23^ gels have provided significant information about the coordination states of Al and the connectivity of Si in these materials. Further to this, solid state MAS NMR experiments probing ^23^Na nuclei have revealed new insight into the charge-balancing role of Na, as well as the incorporation of Sr and Ca, in these materials.^22^ However, MAS NMR spectra of quadrupolar nuclei such as ^39^K and ^87^Sr are much more challenging to measure, particularly in materials with an amorphous nanostructure, and require high magnetic fields not typically available in the laboratory. As such few studies have reported ^39^K MAS NMR data for geopolymers,^24,25^ and to the best of our knowledge none have reported ^87^Sr MAS NMR data for geopolymers. Previous work, however, has reported ^87^Sr MAS NMR data obtained at 21.1 T for SrCO_3_^26,27^ and SrSO_4_^26^ and ^87^Sr MAS NMR data obtained at 7.187 T for Sr(NO_3_)_2_.^28^^87^Sr MAS NMR spectra of inorganic materials such as geopolymers are strongly affected by quadrupolar interactions, with chemical shift distributions contributing insignificantly to the spectral lineshape.^29^

This study examines how different strontium salts influence the reaction mechanisms, K–A–S–H gel nanostructural development, and secondary reaction product formation, in potassium silicate-activated geopolymers. The chemical environments introduced by Sr(OH)_2_·8H_2_O, SrCO_3_, Sr(NO_3_)_2_, and SrSO_4_ simulate the conditions encountered when Sr-bearing waste streams (*e.g.* reactor cooling water, radioactive sludges, slurries, or spent ion-exchange materials) are potentially immobilised within a geopolymer matrix. Fourier transform infrared spectroscopy (FTIR), X-ray diffraction, solid-state magic angle spinning nuclear magnetic resonance (MAS NMR) spectroscopy probing ^27^Al, ^29^Si, ^39^K, and ^87^Sr, synchrotron X-ray absorption near-edge structure (XANES) analysis, and electron probe microanalysis (EPMA) imaging, are used to characterise nanostructural changes induced by the Sr salts, and their influence on Sr incorporation mechanisms. Collectively, the results provide valuable new insight into the nanostructural evolution and Sr incorporation mechanisms in potassium silicate-activated geopolymers, and highlight the suitability of these materials for long-term immobilisation of ^90^Sr-containing radioactive wastes.

## 2 Experimental methods

### 2.1 Sample preparation

Geopolymers were produced by reaction of metakaolin (MetaMax, BASF, UK, chemical composition provided in Table 1, *D*_50_ = 4.49 µm) with a solution of potassium silicate. The activating solution was made by reacting potassium hydroxide (AnalaR 99 wt%), deionized water and a potassium silicate solution (PQ-KS, 51.6% potassium silicate, with a solution modulus of SiO_2_/K_2_O of 2.2, with the balance water, PQ-UK).

**Table 1:** MetaMax metakaolin chemical composition (wt%) as determined by X-ray fluorescence analysis (LOI: loss on ignition at 1000 °C)

SiO_2_	Al_2_O_3_	K_2_O	Na_2_O	MgO	CaO	TiO_2_	Fe_2_O_3_	Other	LOI
52.54	44.54	0.20	0.15	<0.05	<0.05	1.3	0.4	0.2	0.63

Strontium hydroxide octahydrate (Sr(OH)_2_·8H_2_O) (Acros Organics, 98%), strontium carbonate (SrCO_3_) (Sigma Aldrich, >99.9%), strontium nitrate (Sr(NO_3_)_2_) (Alfa Aeser, 98%) or strontium sulphate (SrSO_4_) (Alfa Aeser, Reagent Grade) was added to the samples to achieve a constant Sr/Al molar ratio in the final geopolymer binder (Table 2). The H_2_O/K_2_O ratio for the samples containing Sr(OH)_2_·8H_2_O take into account the water of crystallisation.

**Table 2:** Chemical composition (molar basis), water/solids ratio (w/s; mass basis) of the reaction mixtures

Sample	Anion	Sr/Al	Si/Al	K/Al	H_2_O/K_2_O	w/s
KGP	—	—	1.5	1.0	11	0.55
OH_1	OH^−^	0.025	1.5	1.0	11	0.55
OH_2	OH^−^	0.050	1.5	1.0	11	0.55
OH_3	OH^−^	0.075	1.5	1.0	11	0.54
OH_5	OH^−^	0.125	1.5	1.0	11	0.54

CO_3__1	CO_3_^2−^	0.025	1.5	1.0	11	0.56
CO_3__2	CO_3_^2−^	0.050	1.5	1.0	11	0.57
CO_3__3	CO_3_^2−^	0.075	1.5	1.0	11	0.58

NO_3__1	NO_3_^−^	0.025	1.5	1.0	11	0.56
NO_3__2	NO_3_^−^	0.050	1.5	1.0	11	0.58
NO_3__3	NO_3_^−^	0.075	1.5	1.0	11	0.59
NO_3__5	NO_3_^−^	0.125	1.5	1.0	11	0.62

SO_4__1	SO_4_^2−^	0.025	1.5	1.0	11	0.56
SO_4__2	SO_4_^2−^	0.050	1.5	1.0	11	0.57
SO_4__3	SO_4_^2−^	0.075	1.5	1.0	11	0.57
SO_4__5	SO_4_^2−^	0.125	1.5	1.0	11	0.60

The activating solution was mixed with metakaolin powder and Sr salt with a high-shear mixer at 1300 rpm for 10 minutes, and the geopolymer paste was then cast in sealed containers and cured for 3 and 28 days at 20 °C ± 2 °C. After curing samples were immersed in isopropanol to halt the alkali-activation reaction through the removal of loosely bound water. All characterisation was carried out on samples from the same batch.

### 2.2 Characterisation of the geopolymer samples

#### 2.2.1 X-ray diffraction

X-ray diffraction (XRD) data were obtained across a 2*θ* range of 5°–70° using a Panalytical X'Pert^3^ powder X-ray diffractometer with Cu Kα radiation (1.54 Å), a nickel filter, a step size of 0.020° and a count time of 1 s per step. Diffracted background intensity at low angles was reduced using an anti-scatter blade, an incident beam divergence of 1.0 mm and a 2.5° Soller slit in the diffracted beam were used. Phase identification was performed using the ICDD PDF4+ 2015 database and Diffrac.EVA V4.1 software.

#### 2.2.2 Fourier transform infrared spectroscopy

FTIR spectroscopy data were acquired using a PerkinElmer Frontier Mid FT-IR spectrometer equipped with a deuterated triglycine sulfate (DTGS) detector and KBr beam splitter optical system, scanning 16 times at a resolution of 4 cm^−1^. Data were acquired for pellets comprising 200 mg KBr with 2 mg of sample.

#### 2.2.3 Solid state nuclear magnetic resonance spectroscopy

Solid-state single pulse ^29^Si and ^27^Al magic angle spinning (MAS) nuclear magnetic resonance (NMR) spectra were acquired on a Bruker Avance III HD 500 spectrometer at 11.7 T (*B*_0_) using a 4.0 mm dual resonance CP/MAS probe, yielding a Larmor frequency of 99.35 MHz for ^29^Si and 130.32 MHz for ^27^Al. A measured 60 s relaxation delay, a 5.5 μs non-selective (π/2) excitation pulse, a total of 512 scans and spinning at 12.5 kHz were used to obtain the ^29^Si MAS NMR spectra. ^27^Al MAS NMR spectra were acquired using a 1.7 μs non-selective (π/2) excitation pulse, a measured 10 s relaxation delay, a total of 128 scans and spinning at 12.5 kHz. The same instrument was used to perform the ^29^Si cross-polarisation (CP) MAS NMR experiments, with the parameters differing only through an initial ^1^H non-selective (π/2) pulse width of 2.5 μs, a recycle delay of 1.25 s and Hartmann–Hahn contact periods of 2.0 ms. A nominal ^1^H decoupling field strength of 80 kHz was employed during acquisition and 5120 scans were collected per experiment. The spectra were referenced against pure tetramethylsilane (TMS) at *δ*_iso_ = 0 ppm for ^29^Si and 1.1 M solution Al(NO_3_)_3_ at *δ*_iso_ = 0 ppm for ^27^Al. The deconvolution of the ^29^Si MAS and ^1^H–^29^Si CP MAS NMR spectra was performed using Gaussian peak profiles. For both of the spectral deconvolutions of each resonance, the isotropic chemical shift (*δ*_iso_) and peak full width at half maximum (FWHM) were maintained. Peak intensities were required to be consistent with the structural constraints described by the thermodynamics of a statistical distribution of Si and Al sites within a Q^4^ aluminosilicate network for (N,K)–A–S–H gel products.^30^

High-field solid state ^39^K MAS NMR data were acquired at 20.0 T (*ν*_0_ = 39.67 MHz) using a Bruker Avance Neo 850 spectrometer with a Bruker 4.0 mm HX MAS probe which enabled a spinning rate up to 14 kHz. Pulse calibration and chemical shift referencing for all ^39^K data were achieved using KCl_(s)_ (*δ*_iso_ = 47.8 ppm) as a secondary reference to the IUPAC primary reference of 0.1 M KCl_(aq)_ (*δ*_iso_ = 0 ppm).^31^ A ‘non-selective’ π/2 pulse of 12 μs was measured allowing for a ‘selective’ 4 μs π/2 to be implemented. Spectra were acquired using a double frequency sweep to saturate the central transition followed by a Hahn echo pulse sequence (DFS-p/2–t–p-acquire) using a measured relaxation delay of 0.1 s and acquiring a total of 480 000 scans per spectra. High-field solid state ^87^Sr MAS NMR data were acquired at 20.0 T with a Larmor frequency of 36.843 MHz, using a Bruker Avance Neo 850 spectrometer with a Bruker 4.0 mm HX MAS probe, a rotor synchronised double frequency sweep echo pulse, a measured 0.1 s relaxation delay, spinning at 12.5 kHz, and a total of 839 680 scans. ^87^Sr spectra were referenced against SrTiO_3_ (*δ*_iso_ = 63.4 ppm).^27^

#### 2.2.4 X-ray absorption spectroscopy

XAS data were acquired on the bending magnet beamline B18 at Diamond Light Source, Harwell, UK. Beamline B18 utilises a Pt collimating mirror, a fixed-exit double crystal Si(111) monochromator and a double toroidal focussing mirror. Sr K-edge XAS data were acquired on B18 in transmission mode using finely ground powder specimens dispersed in polyethylene glycol to achieve a thickness of two absorption lengths. Incident and transmitted beam intensities were measured using ionization chambers, filled with mixtures of He and Ar or N_2_, operated in a stable region of their *I*/*V* curve. Yttrium foil was measured to provide an absolute energy calibration; the first inflection point (in the first derivative) was defined to be 17 133 eV. The yttrium foil was measured periodically to ensure that there was no energy drift over the course of the experiment. Data reduction and analysis was performed using the Athena software.^32^

#### 2.2.5 Electron probe microanalysis

Electron probe microanalysis (EPMA) was performed using a Jeol 8350F Plus Hyperprobe equipped with energy dispersive X-ray (EDX) and a wavelength dispersive X-ray (4 channel) system. The samples were analysed using beam conditions of an acceleration voltage of 10 kV and a probe current of 10 nA with a peak coating time of 40 seconds and a measurement of two background (upper and lower) positions for 20 seconds. The intensities of the characteristic X-rays generated by the elements of interest were measured according to their wavelengths by differing crystals, as follows: TAP for Si and Al, PETL for Sr, PETH for Ti and K.

## 3 Results and discussion

### 3.1 Effect of addition of strontium hydroxide octahydrate to geopolymers

#### 3.1.1 Fourier transform infrared spectroscopy

FTIR data for the geopolymers incorporating Sr(OH)_2_·8H_2_O and cured for 3 and 28 days are shown in Fig. S1, SI, and reveal key vibrational bands that provide insight into their chemical structure and reaction. A dominant band at approximately 1000 cm^−1^ corresponds to asymmetric stretching of Si–O–T bonds (T = Si or Al) within the aluminosilicate network of the K–A–S–H gel.^33^ A shoulder at approximately 1050–1100 cm^−1^ indicates the presence of unreacted metakaolin, which has a characteristic band at 1060 cm^−1^.^34^ Additional features include a band at 860 cm^−1^ related to bridging oxygen in AlO_4_ and SiO_4_ groups,^35^ a low-intensity band at 585 cm^−1^ associated with ring structures of TO_4_ units,^36^ and a small peak at 700 cm^−1^ is linked to pseudo-lattice vibrations in aluminosilicate rings.^22^ A low intensity band at 1420 cm^−1^ is attributed to asymmetric stretching vibrations of CO_3_^2−^ anions in SrCO_3_,^37^ due to reaction of atmospheric CO_2_ with excess Sr^2+^_(aq)_.^38^

Comparing the FTIR data for samples with different Sr/Al ratios shows minimal spectral changes, though the shoulder indicating unreacted metakaolin is more intense in the Sr-containing sample (KGP). This suggests enhanced dissolution due to the alkaline and hydrated nature of Sr(OH)_2_·8H_2_O, which raises the solution pH and improves particle wetting. However, previous observations using isothermal calorimetry indicate that this increased dissolution does not enhance gel formation, as higher Sr/Al ratios reduce the total heat released, likely delaying reaction kinetics.^18^ The characteristic Si–O–T band does not shift with Sr addition, implying limited structural incorporation of Sr.^22,25^ After 28 days, FTIR spectra show most bands remain unchanged from day 3, confirming early-stage reaction products shape the final structure. The main differences are a shift in the band due to asymmetric stretching vibrations of CO_3_^2−^ anions in SrCO_3_ (1420 to 1480 cm^−1^) and decreased intensity of the shoulder on the main Si–O–T band due to unreacted metakaolin, indicating continued reaction but no new bond formation.

#### 3.1.2 X-ray diffraction

The X-ray diffraction data for the geopolymer samples containing Sr(OH)_2_·8H_2_O and cured for 3 and 28 days are shown in Fig. S2, SI. The XRD data for each samples cured at 3 and 28 days exhibit a broad feature between 22° 2*θ* and 35° 2*θ*, arising from diffuse scattering and consistent with the presence of a K–A–S–H gel,^39^ as well as reflections due to anatase (TiO_2_, Powder Diffraction file (PDF) # 01-084-1286) which is present as an impurity in the metakaolin used. The presence of strontium hydroxide octahydrate (Sr(OH)_2_·8H_2_O, PDF # 00-027-1438) in samples OH_3 and OH_5 is expected due to the low solubility of the salt,^40^ although these diffraction peaks are not present in the spectra for samples OH_1 and OH_2. This could be due to the concentration being too small in the samples as, potentially, a greater percentage of the mass of the salt has dissolved into the activating solution resulting in a lower mass percentage of solid salt within the sample. The diffraction spectrum for the OH_2 sample shows that there is strontianite (SrCO_3_, PDF # 00-005-0418) present. This is most likely due to the carbonation of the geopolymer during exposure to air, forming SrCO_3_ crystals.

#### 3.1.3 Solid state nuclear magnetic resonance spectroscopy

##### 3.1.3.1 ^29^Si MAS and ^1^H–^29^Si CP MAS NMR

**Fig. 1:**
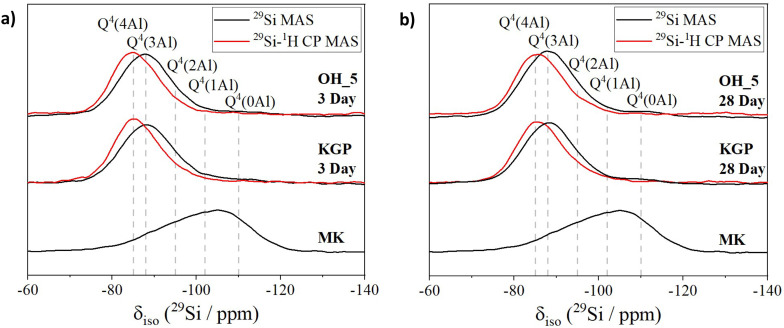
(a) ^1^H–^29^Si CP MAS (shown in red, *B*_0_ = 11.7 T, *ν*_R_ = 12.5 kHz and Hartmann–Hahn contact period *t* = 2.0 ms) and (b) ^29^Si MAS (shown in black, *B*_0_ = 11.7 T, *ν*_R_ = 12.5 kHz) NMR data for geopolymers cured for 3 and 28 days with and without Sr(OH)_2_·8H_2_O, and metakaolin.

The ^1^H–^29^Si CP MAS NMR and ^29^Si MAS NMR data for the OH_5 and KGP samples cured for 3 and 28 days are shown in Fig. 1.

The ^1^H–^29^Si CP MAS NMR data for each geopolymer exhibits a broad resonance centred at *δ*_iso_ = −85.0 ppm and spanning from *δ*_iso_ = −75 to −100 ppm, with a consistent lineshape for all samples. Si sites in the hydrated K–A–S–H gel can be resolved from those in unreacted metakaolin by comparison of the ^1^H–^29^Si CP MAS NMR data with the ^29^Si MAS NMR data. The maximum intensity at *δ*_iso_ = −84.0 ppm in the ^1^H–^29^Si CP MAS NMR spectra of the geopolymer gels, therefore, indicates that the K–A–S–H gel comprises primarily Q^4^(4Al) and Q^4^(3Al) silicon environments.^22,25,41,42^ The data observed here are similar to those seen in previous work, suggesting that the K–A–S–H gel comprises distribution of Q^4^(*m*Al) Si sites, where 1 ≤ *m* ≤ 4.

Deconvolution and quantification of the ^1^H–^29^Si CP MAS and ^29^Si MAS NMR data identifies Q^4^(4Al), Q^4^(3Al), Q^4^(2Al), and Q^4^(1Al) sites within an Al-rich (Si/Al ≤ 1.2), fully polymerised K–A–S–H gel.^20,30^ Engelhardt's formula^43^ (eqn (1)) can be used to calculate the molar Si/Al ratio of the K–A–S–H gel by assuming that there are negligible Al–O–Al bonds present (valid in geopolymers with Si/Al > 1 (ref. 44)), where, *I*_*A*Q^4^(*m*Al)_ represents the normalised relative integral areas of each Q^4^(*m*Al) site within the K–A–S–H gel. The relative integral areas and Si/Al for each sample are shown in Table S1, SI.1
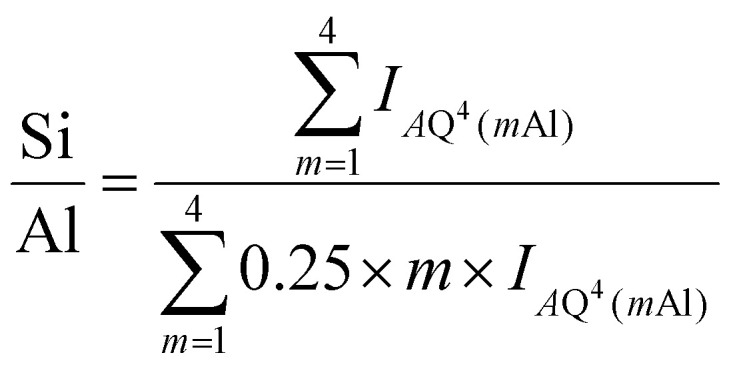


The KGP sample exhibits a slight increase in Si/Al ratio from 1.32 at 3 days to 1.34 at 28 days, supporting the assumption that Al–O–Al bonds are negligible in this system. The OH_5 sample shows consistently higher Si/Al ratios than KGP at both time points. This suggests that the high Sr/Al ratio slightly reduces the gel's ability to incorporate cations, a finding that differs from previous work which reported lower Si/Al ratios in Sr-containing systems.^22^

The elevated Si/Al ratio in the OH_5 sample is attributed to the preferential dissolution of Al_2_O_3_ over SiO_2_ in metakaolin.^19^ Previous work has shown that Sr adsorbs onto the metakaolin surface during reaction, inhibiting the dissolution of Al_2_O_3_ relative to SiO_2_ and increasing the gel Si/Al ratio. The slight decrease in this ratio from day 3 to day 28 in OH_5 implies that metakaolin continues to dissolve over time, consistent with the FTIR data presented above.

##### 3.1.3.2 ^27^Al MAS NMR

**Fig. 2:**
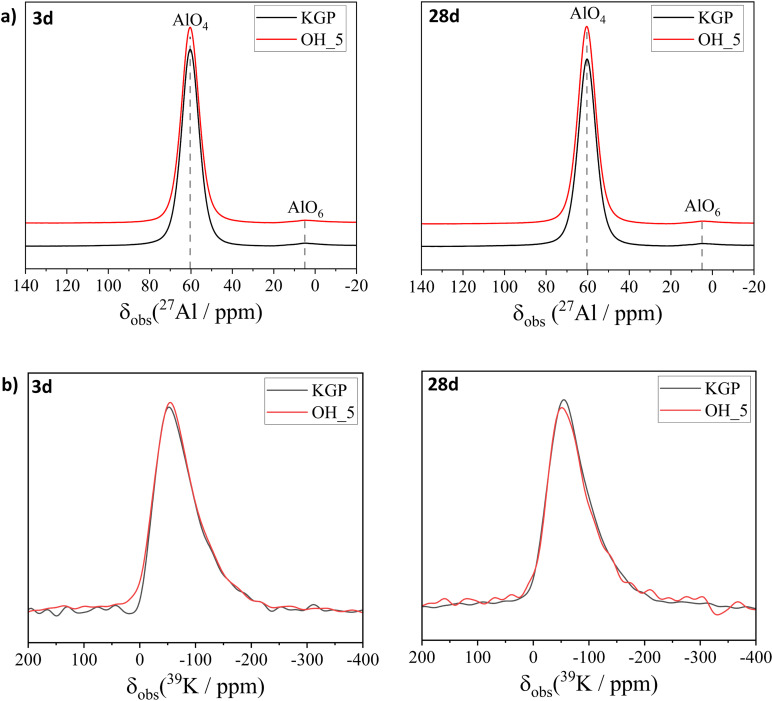
(a) ^27^Al (*B*_0_ = 11.7 T, *ν*_R_ = 12.5 kHz) and (b) ^39^K MAS NMR data (*B*_0_ = 20.0 T, *ν*_R_ = 12.5 kHz) for each geopolymer cured for 3 and 28 days with and without Sr(OH)_2_·8H_2_O, as marked.

^27^Al MAS NMR data for all samples (Fig. 2) exhibit a broad resonance centred at *δ*_obs_ = 60.3 ppm and spanning from *δ*_obs_ = 70 to 50 ppm, which indicates Al within a tetrahedral (q^4^) site in a K–A–S–H type gel,^19,44^ promoted by the significant excess of alkali cations able to balance the negative charge due to Al^3+^ in tetrahedral coordination. The preference of Al to exist within the q^4^(4Si) coordination is due to the energetic penalty associated with Al–O–Al bonding.^30,45^ A low intensity resonance observed at *δ*_obs_ = 5 ppm is attributed to the presence of octahedral Al sites (AlO_6_) within the samples.^46^ The intensity of this peak is negligible when compared to that of the tetrahedral Al peak, and suggests that the majority of the Al within the metakaolin has reacted as the octahedral peak corresponds exclusively to the Al found within the unreacted metakaolin.^19^ This is supported by the findings of both the ^29^Si MAS and ^1^H–^29^Si CP MAS NMR data which display the preferential dissolution of Al during the dissolution phase of the reaction. No differences in either resonance are observed when comparing samples with and without Sr.

##### 3.1.3.3 ^39^K MAS NMR

The ^39^K MAS NMR data (Fig. 2) for the geopolymer samples formulated with and without Sr(OH)_2_·8H_2_O each exhibit a broad resonance spanning from *δ* = 0 ppm to −220 ppm, centred at *δ* = −50 ppm for geopolymers incorporating Sr(OH)_2_·8H_2_O and *δ* = −55 ppm for the KGP samples. The resonance exhibits a broad asymmetric lineshape which is characteristic of a quadrupolar nucleus in a disordered material. This resonance is attributed to charge-balancing extra-framework K^+^ ions within the K–A–S–H-type gel,^24,25^ and the line shape of the data for all samples is comparable, suggesting little change to the chemical environments experienced by the K atoms within the sample. The difference in chemical shift observed in the data here is consistent with the shielding of K^+^ cations due to the presence of Sr^2+^ in the charge balancing sites within close proximity to K, consistent with incorporation of Sr^2+^ into the K–A–S–H gel to form a K–(Sr)–A–S–H gel.

##### 3.1.3.4 ^87^Sr MAS NMR

**Fig. 3:**
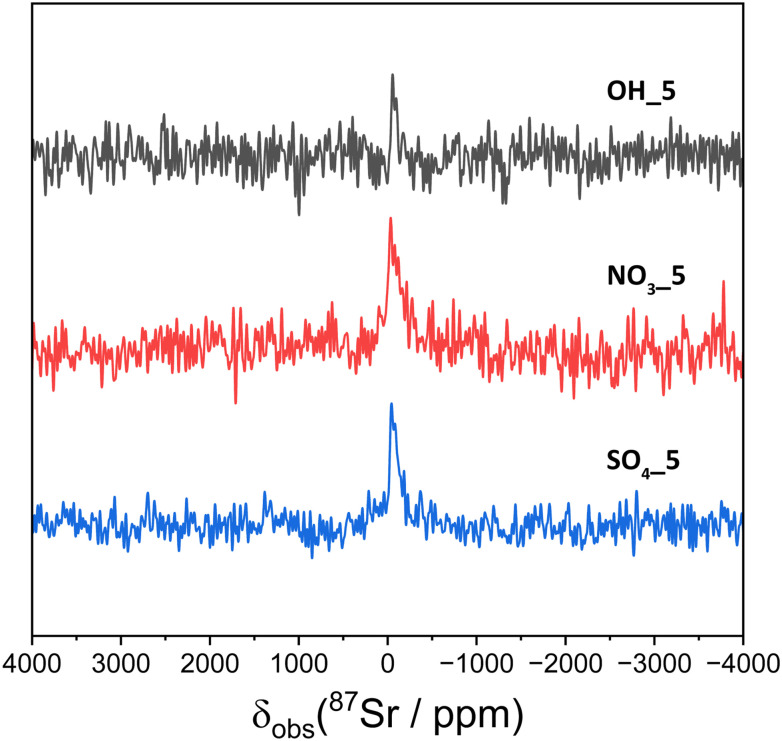
^87^Sr MAS NMR data (*B*_0_ = 20.0 T, *ν*_R_ = 12.5 kHz, with 1024 data points transformed (out of 8192 data points obtained) and line broadening of 100 Hz applied) for the OH_5, NO3_5 and SO4_5 geopolymer samples cured for 3 days, showing the full spectral width acquired.

The ^87^Sr MAS NMR data for the OH_5 sample after curing for 3 days are shown in Fig. 3, across the full spectral width obtained, along with the ^87^Sr MAS NMR data for the NO_3__5 and SO_4__5 samples for comparison (despite numerous attempts ^87^Sr MAS NMR data was not able to be obtained for the CO_3__5 sample). The ^87^Sr NMR data each of these samples shows a single resonance with a broad quadrupolar lineshape centred at ∼*δ*_obs_ = −45 ppm to −65 ppm, with a full width at half maximum (FWHM) on the order of ∼5 kHz. ^87^Sr MAS NMR data are very difficult to obtain,^29^ particularly for disordered materials, however the data obtained here exhibit chemical shifts and FWHM that are broadly consistent with ^87^Sr MAS NMR data obtained previously for Sr(NO_3_)_2_, SrSO_4_, and strontium silicate compounds.^28,29^

**Fig. 4:**
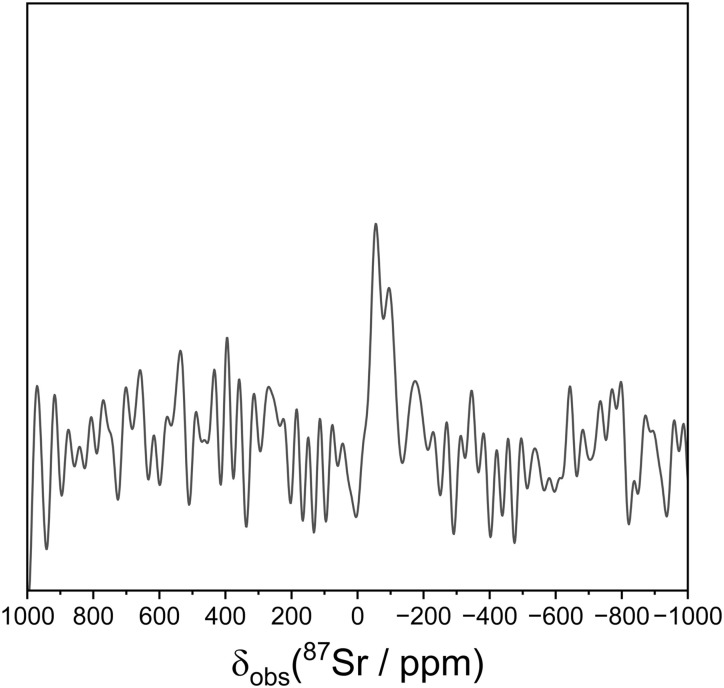
^87^Sr MAS NMR data (*B*_0_ = 20.0 T, *ν*_R_ = 12.5 kHz, with 1024 data points transformed (out of 8192 data points obtained) and line broadening of 100 Hz applied) for the OH_5 geopolymer sample.

The ^87^Sr NMR data for the OH_5 sample after curing for 3 days are shown in Fig. 4 across a smaller spectral width focused on the obtained signal. The data exhibit a single resonance with a broad quadrupolar lineshape, centred at *δ*_obs_ = −63 ppm. The low solubility of Sr(OH)_2_·8H_2_O in the activating solution, and observations in the XRD data discussed above, suggest that this is potentially due to Sr(OH)_2_·8H_2_O.^18^ The crystalline structure of Sr(OH)_2_ is tetragonal, where each Sr atom is surrounded by 8 water molecules.^47,48^ The central Sr^2+^ ion is coordinated into an antiprism configuration by 8 oxygen (O) atoms by the H_2_O molecules.^49^ If the Sr^2+^ cations in these samples are bonded into the geopolymer, it is likely that the formation of a pseudo-zeolite will occur, similar to that of brewsterite, a natural strontium containing zeolite.^25^ This hypothesis is supported by the literature, suggesting that similar zeolitic phases form within geopolymer structures after the inclusion of different cations.^50,51^ The structure of this is chemically distinct to that of the crystalline Sr(OH)_2_, where each of the Sr atoms are bonded to four framework O atoms and five H_2_O molecules.^52,53^ Additionally, if Sr^2+^ cations in these samples are bonded into the geopolymer aluminosilicate framework they are expected to exist in a brewsterite-type local structure,^25,50,51^ which may exhibit some similarities in shielding of the ^87^Sr nuclei to the SrSiO_3_ phase observed in nuclear waste glasses. The latter has been observed to exhibit an ^87^Sr MAS NMR resonance at *δ*_iso_ = 0 ppm.^54^ The ^87^Sr MAS NMR data shown in Fig. 4 do not exhibit intensity in this region, and no additional resonances are clearly observed in the data.

#### 3.1.4 X-ray absorption near-edge structure (XANES) spectroscopy

**Fig. 5:**
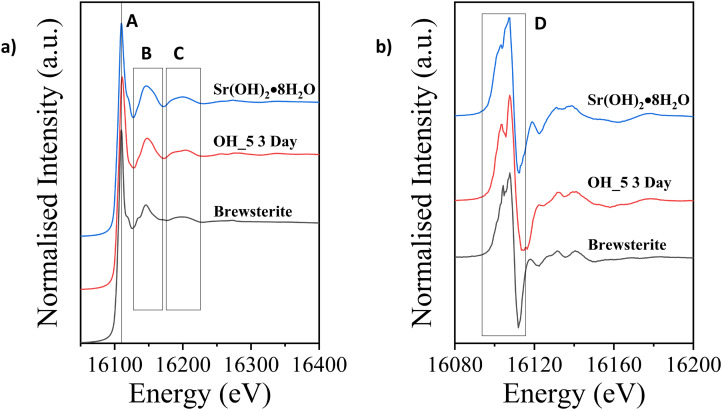
(a) Normalised X-ray absorption spectra for the geopolymer samples cured for 3 days with Sr(OH)_2_·8H_2_O, the Sr(OH)_2_·8H_2_O reactant used, and the mineral brewsterite-Sr, and (b) the 1^st^ derivative of these spectra.

The normalised spectra for the Sr K-edge XAS analysis of the OH_5 sample after 3 days of reaction compared to both the Sr(OH)_2_·8H_2_O reagent and the mineral brewsterite-Sr ((Sr,Ba)_2_Al_4_Si_12_O_32_·10H_2_O) are shown in Fig. 5.^53^

The Sr K-edge is relatively featureless, but the highlighted regions in Fig. 5 allow for some qualitative analysis between samples.^55–57^ When inspecting Fig. 5b, the presence of the doublet on the rising edge is observed. This feature is highlighted by the box labelled D in Fig. 5b, and is far more pronounced in the brewsterite-Sr mineral than in the Sr(OH)_2_·8H_2_O reagent. Additionally, when comparing this to the OH_5 sample, the peak is more resembling of the brewsterite spectra than the Sr(OH)_2_·8H_2_O salt, but still displays features of both, most notably the difference in maximums between the two peaks which is more resemblant of the salt. To build upon this, referring back to Fig. 5a, the OH_5 sample appears to exhibit further features of both the salt and Sr in a pseudo-zeolitic structural environment. When exploring the features above the absorption edge, it can be seen that within feature B, the OH_5 sample appears to have a small shoulder at ∼16 135 eV, which is at the same energy as that of the shoulder in the brewsterite spectra. However, this exact same peak follows a similar path to that of the corresponding peak in the Sr(OH)_2_·8H_2_O spectra. Furthermore, feature C for the OH_5 sample appears to show features relating to both the Sr(OH)_2_·8H_2_O reagent and brewsterite, with the lower energy region of this area following a curve comparable to that of brewsterite and the higher energy region comparing to the Sr(OH)_2_·8H_2_O reagent.

From this data, it is implied that the OH_5 sample contains Sr environments both in pseudo-zeolitic structural environments, *i.e.* incorporated as the charge balancing cation in the K–A–S–H gel, and also in the structural environment of Sr(OH)_2_·8H_2_O. This finding is supported by the Sr NMR data, which suggests that there are two Sr environments present within the sample.

#### 3.1.5 Electron probe microanalysis

**Fig. 6:**
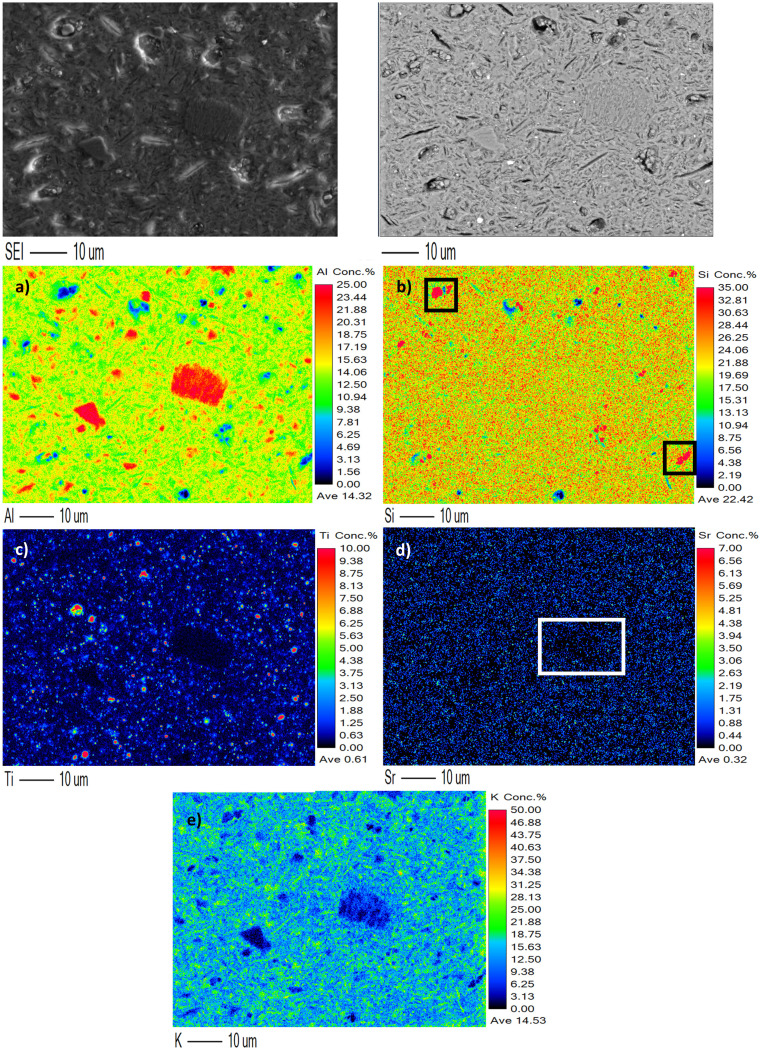
Electron probe microanalysis images generated for the OH_5 geopolymer for 28 days showing distribution of (a) aluminium, (b) silicon, (c) titanium, (d) strontium, and (e) potassium within the sample.

EPMA images of the OH_5 sample cured for 28 days are shown in Fig. 6, and the data exhibit a distribution of Al, Si, and K consistent with the amorphous K–A–S–H gel framework. Unreacted metakaolin particles are observed as Al-rich (dark red) areas, often correlating with Ti-rich regions arising due to the presence of anatase as an impurity in the metakaolin used (identified by XRD and discussed above). Porosity is visible as dark blue regions. Sr appears uniformly distributed as small discrete particles, suggesting it does not dissolve fully in the activating solution and acts primarily as a filler in the geopolymer sample, becoming physically embedded within the matrix. This supports previous observations using isothermal calorimetry and FTIR, which found that the low solubility of Sr(OH)_2_·8H_2_O in the alkali activating solution resulted in limited dissolution of the salt.^18^ Some regions have slightly higher Sr concentration, likely due to larger salt particles. K occupies charge-balancing sites within the alkali aluminosilicate framework, and is distributed widely throughout the sample, with regions exhibiting low concentration of K aligning with regions attributed to unreacted metakaolin particles.

### 3.2 Effect of addition of strontium carbonate to geopolymers

#### 3.2.1 Fourier transform infrared spectroscopy

FTIR data for the geopolymers incorporating SrCO_3_ and cured for 3 and 28 days are shown in Fig. S3, SI. The data are very similar to those obtained for geopolymers incorporating Sr(OH)_2_·8H_2_O, with a dominant band due to asymmetric stretching of Si–O–T bonds (T = Si or Al) within the K–A–S–H gel.^33^ A shoulder at approximately 1050–1100 cm^−1^ indicates the presence of unreacted metakaolin, and bands at 1480 cm^−1^, 700 cm^−1^, and 855 cm^−1^ arise due to asymmetric stretching vibrations of CO_3_^2−^ anions in SrCO_3_,^37^ indicating the presence of SrCO_3_ within the geopolymer after 28 days of reaction. Comparing the FTIR data for samples with different Sr/Al ratios, the intensity of the bands arising due to the presence of SrCO_3_ increase with increasing Sr/Al ratio, corroborating this assignment. This indicates that SrCO_3_ does not dissolve fully within the activating solution and resides in its solid form within the pores of the gel; SrCO_3_ has a solubility limit of 0.00034 g per 100 g of H_2_O and this reduces as alkalinity increases.^58^ The characteristic Si–O–T band does not shift with Sr addition, implying limited structural incorporation of Sr.^22,25^ After curing for 28 days, the main difference in the data compared to that for the sample cured for 3 days is the decreased intensity of the shoulder on the main Si–O–T band due to unreacted metakaolin, indicating continued reaction but no new bond formation.

#### 3.2.2 X-ray diffraction

The X-ray diffraction data for the geopolymer samples containing SrCO_3_ and cured for 3 and 28 days are shown in Fig. S4, SI. The data show the same features as the XRD data for the geopolymer samples containing Sr(OH)_2_·8H_2_O, with a broad feature between 22° 2*θ* and 35° 2*θ*, arising from diffuse scattering and consistent with the presence of a K–A–S–H gel,^39^ as well as reflections due to anatase (TiO_2_, PDF # 01-084-1286) which is present as an impurity in the metakaolin used, and reflections due to strontianite (SrCO_3_, PDF # 00-005-0418). This is consistent with the FTIR data for these samples, and corroborates the interpretation that SrCO_3_ exhibits low solubility in these samples.

#### 3.2.3 Solid state nuclear magnetic resonance spectroscopy

##### 3.2.3.1 ^29^Si MAS and ^1^H–^29^Si CP MAS NMR

**Fig. 7:**
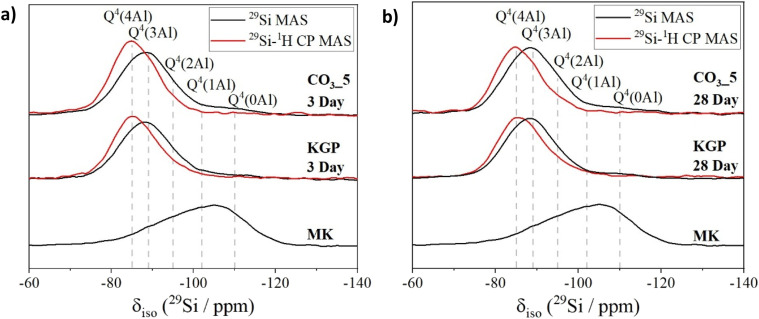
(a) ^1^H–^29^Si CP MAS (shown in red, *B*_0_ = 11.7 T, *ν*_R_ = 12.5 kHz and Hartmann–Hahn contact period *t* = 2.0 ms) and (b) ^29^Si MAS (shown in black, *B*_0_ = 11.7 T, *ν*_R_ = 12.5 kHz) NMR data and associated deconvolutions for geopolymers cured for 3 and 28 days with and without SrCO_3_, and metakaolin.

The ^1^H–^29^Si CP MAS NMR and ^29^Si MAS NMR data for the CO_3__5 and KGP samples cured for 3 and 28 days are shown in Fig. 7. The ^1^H–^29^Si CP MAS NMR data for each geopolymer exhibits a broad resonance centred at *δ*_iso_ = −85.0 ppm and spanning from *δ*_iso_ = −75 to −100 ppm, with a consistent lineshape for all samples. Deconvolution and quantification of the ^1^H–^29^Si CP MAS and ^29^Si MAS NMR data (Table S2, SI) identifies Q^4^(4Al), Q^4^(3Al), Q^4^(2Al), and Q^4^(1Al) sites within an Al-rich (Si/Al ≤ 1.2), fully polymerised K–A–S–H gel,^20,30^ with Si/Al ratios similar to that observed for geopolymers incorporating Sr(OH)_2_·8H_2_O. The CO_3__5 sample shows consistently higher Si/Al ratios than KGP at both time points, which may arise from a lower extent of reaction and hence greater proportion of Si sites from unreacted metakaolin in these samples compared to the control samples. This is consistent with the FTIR and XRD data for these samples, as well as previous observations by isothermal calorimetry.^18^ The addition of SrCO_3_ to the geopolymer system reduces both the reaction rate and the extent of reaction (*i.e.*, the total heat evolved is lower, as observed previously by isothermal calorimetry) compared to the control geopolymer sample. Because SrCO_3_ is poorly soluble under the high-pH conditions used, many particles remain undissolved and therefore act like an inert filler. This means there is effectively less reactive material (metakaolin + activator) per unit volume, and the reactive surface area is reduced. This reduces the rate of reaction. The calculated extent of reaction shows that the metakaolin precursor continues to react between 3 and 28 days curing.

##### 3.2.3.2 ^27^Al and ^39^K MAS NMR

**Fig. 8:**
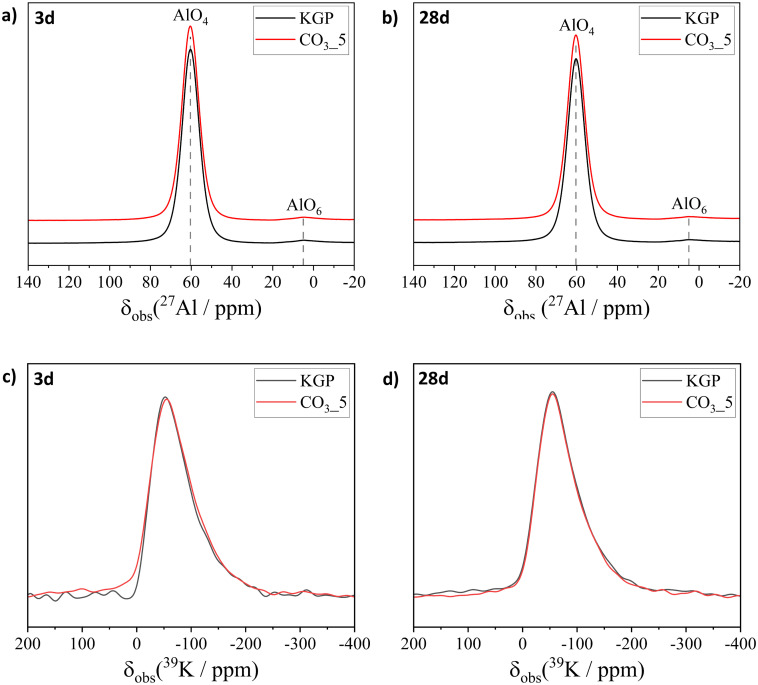
(a) ^27^Al (*B*_0_ = 11.7 T, *ν*_R_ = 12.5 kHz) and (b) ^39^K MAS NMR data (*B*_0_ = 20.0 T, *ν*_R_ = 12.5 kHz) for each geopolymer cured for with and without SrCO_3_.

^27^Al and ^39^K MAS NMR data for all samples (Fig. 8) are nearly identical to those obtained for geopolymers samples incorporating Sr(OH)_2_·8H_2_O, indicating that Al exists within tetrahedral (q^4^) sites in the K–A–S–H type gel as well as octahedral Al sites within remnant unreacted metakaolin, and K exists in charge-balancing extra-framework sites within the K–A–S–H-type gel.^19,44^ This is supported by the findings of both the ^29^Si MAS and ^1^H–^29^Si CP MAS NMR data for these samples. No differences in either resonance are observed when comparing samples with and without Sr.

#### 3.2.4 X-ray absorption near-edge structure (XANES) spectroscopy

**Fig. 9:**
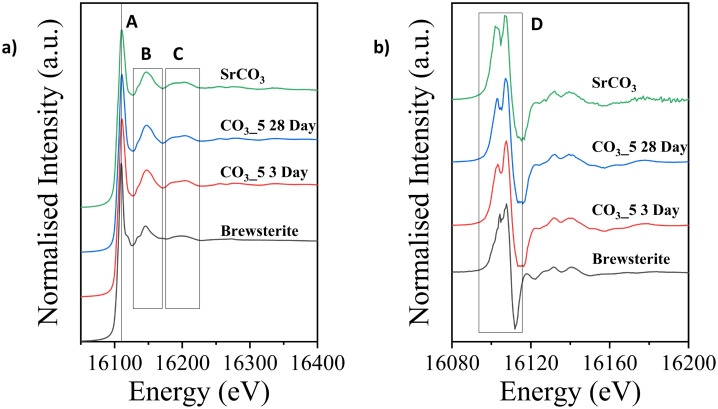
(a) Normalised X-ray absorption spectra for the geopolymer samples loaded with SrCO_3_, reagent grade SrCO_3_, and the mineral brewsterite-Sr, and (b) the 1^st^ derivative of these spectra.

The normalised spectra for the Sr K-edge XAS analysis of the CO_3__5 sample after 3 and 28 days of reaction compared to both the SrCO_3_ reagent and brewsterite-Sr ((Sr,Ba)_2_Al_4_Si_12_O_32_·10H_2_O) are shown in Fig. 9.^53^ The Sr K-edge here displays an absorption edge that rises smoothly to a maximum featuring a singular peak. The Sr K-edge is relatively featureless but provides enough information for a qualitative analysis to be performed. Firstly, the peak maximum, labelled A in Fig. 9a, of the brewsterite spectra is reached at slightly lower energies to that of the SrCO_3_ reagent and the CO_3__5 samples at both time points. This peak appears to be singular in this figure, however, the utilisation of the 1^st^ derivative figure, shown in Fig. 9b, shows that there is a very shallow shoulder on the edge. The highlighted region of Fig. 9b, labelled D, shows that this doublet peak in the 1^st^ derivative data is more pronounced in the SrCO_3_ when compared to the brewsterite, and that both of the CO_3__5 samples show similarities with the SrCO_3_ salt spectra in this region. In addition, referring back to Fig. 9a, the features in both highlighted region B and C appear to confirm that the majority of the Sr environments within the CO_3__5 samples at both time points correspond to those of the Sr in SrCO_3_. There does not seem to be any difference between the spectra for the CO_3__5 samples at 3 and 28 days post reaction. The qualitative analysis performed here indicates that the Sr resides within the sample in the form SrCO_3_. This is supported by FTIR, XRD, and MAS NMR data discussed above.

#### 3.2.5 Electron probe microanalysis

**Fig. 10:**
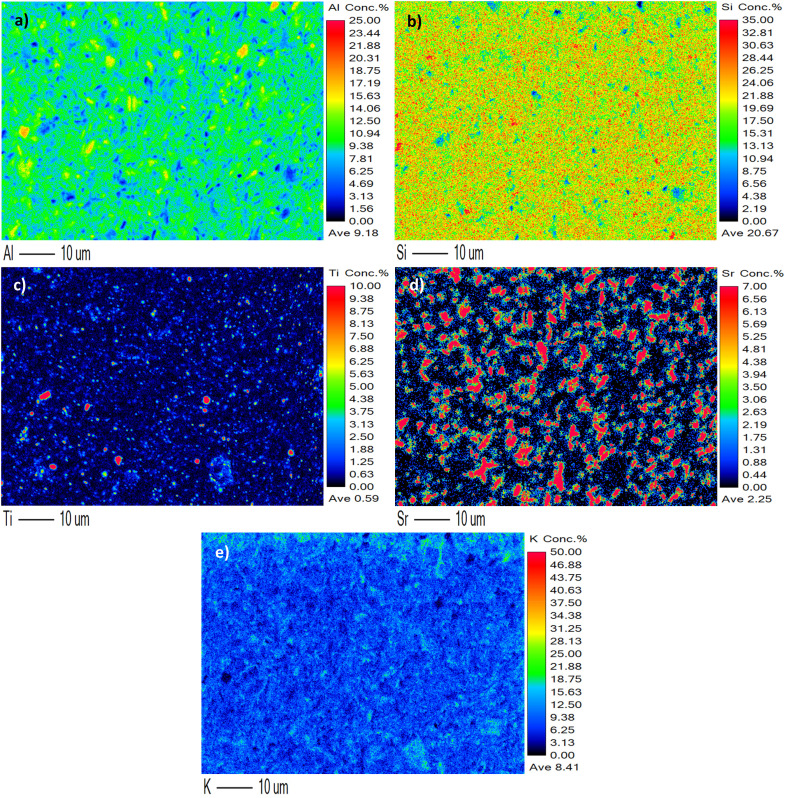
Electron probe microanalysis images generated for the CO_3__5 geopolymer for 28 days showing distribution of (a) aluminium, (b) silicon, (c) titanium, (d) strontium, and (e) potassium within the sample.

EPMA images of the CO_3__5 sample cured for 28 days are shown in Fig. 10, and the data exhibit a distribution of Al, Si, and K consistent with the amorphous K–A–S–H gel framework. Similar to the data for geopolymers incorporating Sr(OH)_2_·8H_2_O, the data show unreacted metakaolin particles (observed as Al-rich regions), also correlating with Ti-rich regions arising due to the presence of anatase as an impurity in the metakaolin used. Sr is located in discrete particles or clusters of particles a few microns in diameter, contrasting with the observations for geopolymers incorporating of Sr(OH)_2_·8H_2_O which showed Sr was uniformly distributed throughout the samples is sub-micron particles. This indicates that Sr in geopolymers incorporating of SrCO_3_ exists in discrete SrCO_3_ particles, consistent with its low solubility within these samples. K occupies charge-balancing sites within the alkali aluminosilicate framework, and is distributed widely throughout the sample, with regions exhibiting low concentration of K align with regions attributed to unreacted metakaolin particles.

### 3.3 Effect of addition of strontium nitrate to geopolymers

#### 3.3.1 Fourier transform infrared spectroscopy

FTIR data for the geopolymers incorporating Sr(NO_3_)_2_ and cured for 3 and 28 days are shown in Fig. S5, SI. The data are very similar to those obtained for geopolymers incorporating Sr(OH)_2_·8H_2_O and SrCO_3_, with a dominant band due to asymmetric stretching of Si–O–T bonds (T = Si or Al) within the K–A–S–H gel^33^ and a shoulder at approximately 1050–1100 cm^−1^ indicating the presence of unreacted metakaolin. Vibrational bands at 1385 cm^−1^ and at 825 cm^−1^ are attributed to vibrations in NO_3_^−^ ions in Sr(NO_3_)_2_ and KNO_3_, indicating that Sr(NO_3_)_2_ has dissolved to some extent, and the subsequent reaction in the aqueous phase has resulted in formation and precipitation of KNO_3_, and that some unreacted Sr(NO_3_)_2_ is also present within these samples after 28 days of reaction. Comparing the FTIR data for samples with different Sr/Al ratios, the intensity of the bands arising due to the presence of Sr(NO_3_)_2_ and KNO_3_ increase with increasing Sr/Al ratio, corroborating this assignment.

#### 3.3.2 X-ray diffraction

The X-ray diffraction data for the geopolymer samples containing Sr(NO_3_)_2_ and cured for 3 and 28 days are shown in Fig. S6, SI. The data show a broad feature between 22° 2*θ* and 35° 2*θ*, arising from diffuse scattering and consistent with the presence of a K–A–S–H gel,^39^ as well as reflections due to anatase (TiO_2_, PDF # 01-084-1286) which is present as an impurity in the metakaolin used. The data also contain reflections due to niter (KNO_3_, PDF # 01-071-1558) suggests that the NO_3_^−^ ions in solution due to the dissolution of the Sr(NO_3_)_2_ are reacting with the aqueous K^+^ ions forming KNO_3_. The observation of low intensity reflections due to strontianite (SrCO_3_, PDF # 00-005-0418) indicates that some of the dissolved Sr^2+^ is reacting with dissolved carbonic acid (H_2_CO_3_), itself arising from dissolution of atmospheric CO_2_ in the pore solution. Together with the FTIR data for these samples, this shows that Sr(NO_3_)_2_ is significantly soluble in the fresh state aqueous phase of these samples.

#### 3.3.3 Solid state nuclear magnetic resonance spectroscopy

##### 3.3.3.1 ^29^Si MAS and ^1^H–^29^Si CP MAS NMR

**Fig. 11:**
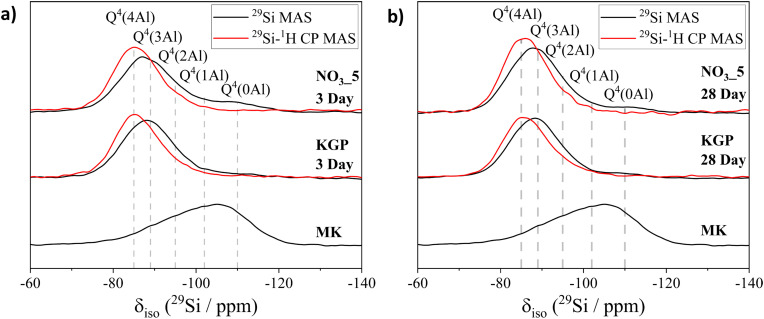
(a) ^1^H–^29^Si CP MAS (shown in red, *B*_0_ = 11.7 T, *ν*_R_ = 12.5 kHz and Hartmann–Hahn contact period *t* = 2.0 ms) and (b) ^29^Si MAS (shown in black, *B*_0_ = 11.7 T, *ν*_R_ = 12.5 kHz) NMR data and associated deconvolutions for geopolymers cured for 3 and 28 days with and without Sr(NO_3_)_2_, and metakaolin.

The ^1^H–^29^Si CP MAS NMR and ^29^Si MAS NMR data for the NO_3__5 and KGP samples cured for 3 and 28 days are shown in Fig. 11. The ^1^H–^29^Si CP MAS NMR data for each geopolymer exhibits a broad resonance centred at *δ*_iso_ = −85.0 ppm and spanning from *δ*_iso_ = −75 to −100 ppm, with a consistent lineshape for all samples. Deconvolution and quantification of the ^1^H–^29^Si CP MAS and ^29^Si MAS NMR data identifies Q^4^(4Al), Q^4^(3Al), Q^4^(2Al), and Q^4^(1Al) sites within an Al-rich (Si/Al ≤ 1.2), fully polymerised K–A–S–H gel,^20,30^ with Si/Al ratios similar to that observed for geopolymers incorporating Sr(OH)_2_·8H_2_O and SrCO_3_. The NO_3__5 sample appears to have a gel structure dominated by Q^4^(4Al), Q^4^(3Al) and Q^4^(2Al) silicon sites after 3 days but after 28 days, the percentage of Q^4^(2Al) sites drops significantly to a structure dominated by Q^4^(4Al) and Q^4^(3Al) sites. This shows that the Al content of the K–A–S–H gel is increasing over time, agreeing with the Si/Al ratios shown in Table S3, SI. Previous work using zeta potential measurements of dispersions of metakaolin particles in solutions representative of the aqueous phase in the fresh geopolymers studied in this work suggest that the dissolution of the metakaolin is slowed due to the adsorption of Sr^2+^ cations to its surface, reducing electrostatic repulsion resulting in particle agglomeration (and hence a lower reactive surface area), forming a Sr^2+^ layer on the surface that physically and electrostatically shields metakaolin from OH^−^ ions, reducing dissolution of Al/Si species needed to form the K–A–S–H gel.^18^ The results here show that this leads to a Si-rich K–A–S–H gel in the early stages of the reaction (due to the presence of excess soluble silica from the activating solution) which becomes more Al-rich as the sample ages. This is consistent with the FTIR and XRD data for these samples, as well as previous observations by isothermal calorimetry.^18^ The calculated extent of reaction shows that the metakaolin precursor continues to react between 3 and 28 days curing, however these are lower than when geopolymers are produced in the presence of Sr(OH)_2_·8H_2_O and SrCO_3_, consistent with the lower rate of reaction due to the presence of Sr on the surface of metakaolin particles described above (Fig. 11).^18^

##### 3.3.3.2 ^27^Al and ^39^K MAS NMR

**Fig. 12:**
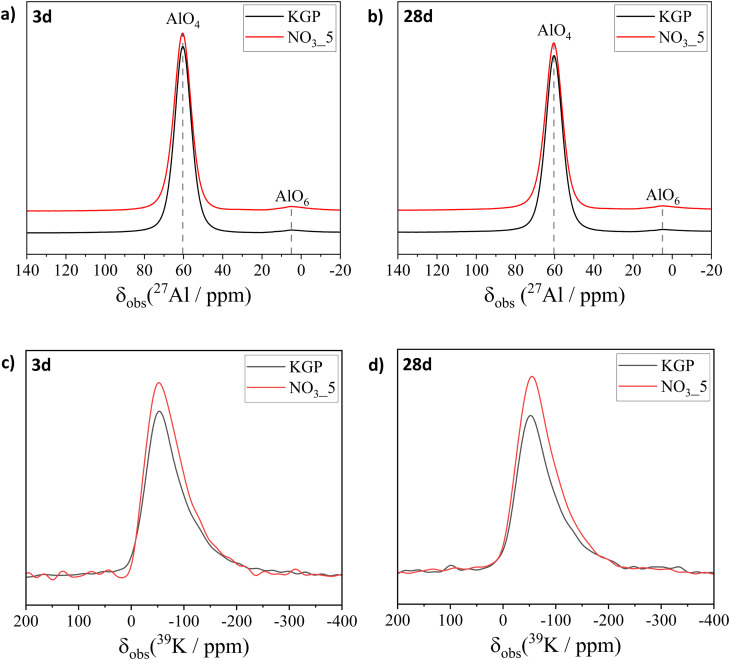
(a) ^27^Al (*B*_0_ = 11.7 T, *ν*_R_ = 12.5 kHz) and (b) ^39^K MAS NMR data (*B*_0_ = 20.0 T, *ν*_R_ = 12.5 kHz) for each geopolymer cured for 3 and 28 days with and without Sr(NO_3_)_2_.

^27^Al and ^39^K MAS NMR data for all samples (Fig. 12) are nearly identical to those obtained for geopolymers samples incorporating Sr(OH)_2_·8H_2_O and SrCO_3_, indicating that Al exists within tetrahedral (q^4^) sites in the K–A–S–H type gel as well as octahedral Al sites within remnant unreacted metakaolin, and K exists in charge-balancing extra-framework sites within the K–A–S–H-type gel.^19,44^ This is supported by the findings of both the ^29^Si MAS and ^1^H–^29^Si CP MAS NMR data for these samples. No differences in either resonance are observed when comparing samples with and without Sr.

**Fig. 13:**
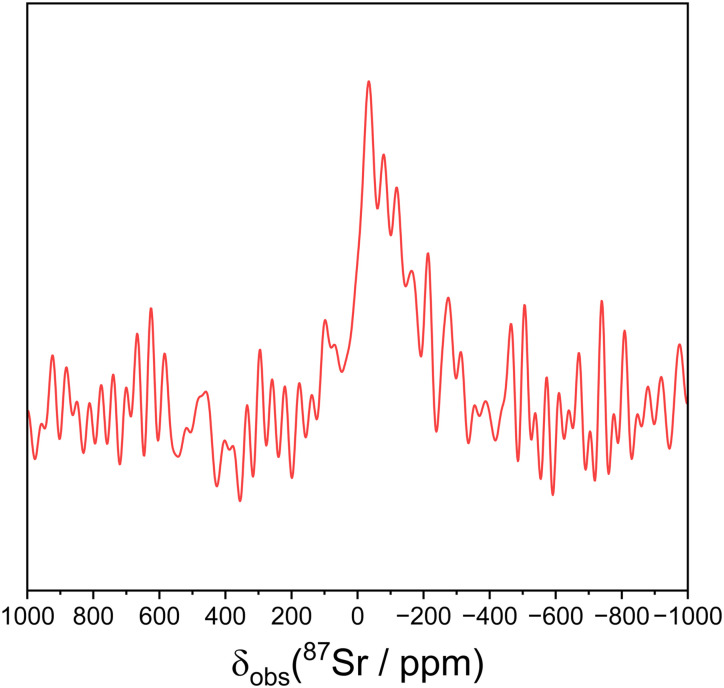
^87^Sr MAS NMR data (*B*_0_ = 20.0 T, *ν*_R_ = 12.5 kHz with 1024 data points transformed (out of 8192 data points obtained) and line broadening of 100 Hz applied) for the NO_3__5 geopolymer sample cured for 3 days with Sr(NO_3_)_2_.

The ^87^Sr MAS NMR data for the NO_3__5 sample after curing for 3 days are shown in Fig. 13. Similar to the data obtained for geopolymers incorporating Sr(OH)_2_·8H_2_O, the ^87^Sr MAS NMR data for the NO_3__5 sample after curing for 3 days exhibits a single distinct resonance with a broad quadrupolar lineshape, centred at approximately *δ*_obs_ = −73 ppm. This is broadly consistent with the chemical shift and FWHM observed for ^87^Sr MAS NMR data for Sr(NO_3_)_2_,^28^ suggesting that this phase may be responsible for the resonance observed in the data here. Additionally, as discussed above, if Sr^2+^ cations in these samples are bonded into the geopolymer aluminosilicate framework they are expected to exist in a brewsterite-type local structure,^25,50,51^ which may exhibit some similarities in shielding of the ^87^Sr nuclei to the SrSiO_3_ phase observed in nuclear waste glasses.^54^ The latter has been observed to exhibit an ^87^Sr MAS NMR resonance at *δ*_iso_ = 0 ppm. The ^87^Sr MAS NMR data shown in Fig. 13 exhibit significant intensity in this region, as a shoulder on the main resonance, however this shoulder is not significantly above the spectral noise, and so no firm conclusions can be drawn from this data about the existence of this site.

#### 3.3.4 X-ray absorption near-edge structure (XANES) spectroscopy

**Fig. 14:**
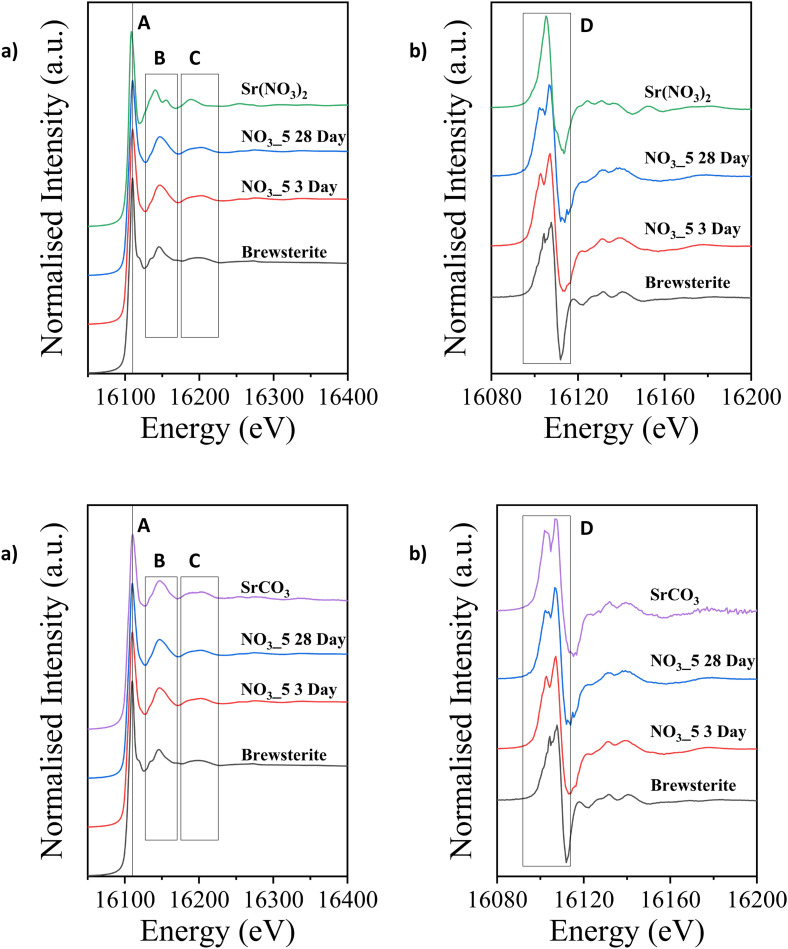
(a) Normalised X-ray absorption spectra for the geopolymer samples loaded with Sr(NO_3_)_2_, reagent grade Sr(NO_3_)_2_, and the mineral brewsterite-Sr, and (b) the 1^st^ derivative of these spectra.

The normalised spectra for the Sr K-edge XAS analysis of the NO_3__5 sample after 3 and 28 days of reaction compared to both the Sr(NO_3_)_2_ reagent and brewsterite-Sr ((Sr,Ba)_2_Al_4_Si_12_O_32_·10H_2_O) are shown in Fig. 14.^53^ The XRD data for these samples show that there is a quantity of SrCO_3_ within the NO_3__5 sample, and so it may be more relevant to compare the XANES spectra to a SrCO_3_ standard (Fig. 14). Firstly, by inspecting the region labelled as B in Fig. 14a, the line shape of the spectra for both NO_3__5 samples aligns more closely with the line shape of the SrCO_3_ standard as opposed to the brewsterite. The shoulder on the peak that is easily seen in the brewsterite spectra cannot be seen so clearly in the spectra for the NO_3__5 samples. Moreover, the feature in region C aligns closely between the SrCO_3_ and the NO_3__5 samples. The evidence here suggest that the Sr is likely to be mainly found in the form of SrCO_3_, with smaller quantities of Sr present in a brewsterite-type local structure. Overall, the spectra here suggest that all of the Sr(NO_3_)_2_ dissolves within the activating solution upon its initial addition. The Sr^2+^ cations that are subsequently released into solution are likely to react with the CO_2_ in the air to form crystalline SrCO_3_, which is supported by the XRD and the XANES spectra here.

#### 3.3.5 Electron probe microanalysis

**Fig. 15:**
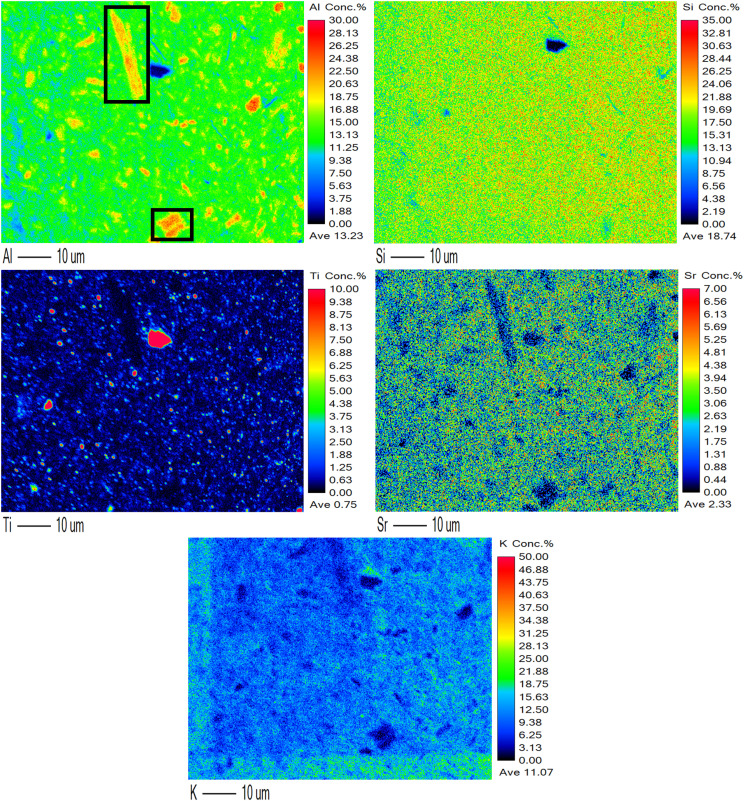
Electron probe microanalysis images generated for the NO_3__5 geopolymer for 28 days showing distribution of (a) aluminium, (b) silicon, (c) titanium, (d) strontium, and (e) potassium within the sample.

EPMA images of the NO_3__5 sample cured for 28 days are shown in Fig. 15, and the data exhibit a distribution of Al, Si, and K consistent with the amorphous K–A–S–H gel framework. Similar to the data for geopolymers incorporating of Sr(OH)_2_·8H_2_O, the data show unreacted metakaolin particles (observed as Al-rich regions), also correlating with Ti-rich regions arising due to the presence of anatase as an impurity in the metakaolin used. Sr appears uniformly distributed throughout the geopolymer matrix, supporting the possibility that Sr^2+^ is displacing K^+^ to act as a charge-balancing cation within the K–A–S–H gel framework (also indicated by XANES data). Furthermore, the concentration of K is also distributed evenly throughout the geopolymer matrix, suggesting that K remains the primary charge-balancing cation.

### 3.4 Effect of addition of strontium sulfate to geopolymers

#### 3.4.1 Fourier transform infrared spectroscopy

FTIR data for the geopolymers incorporating SrSO_4_ and cured for 3 and 28 days are shown in Fig. S7, SI. The data are similar to those obtained for geopolymers incorporating Sr(OH)_2_·8H_2_O, SrCO_3_, and Sr(NO_3_)_2_, with a dominant band due to asymmetric stretching of Si–O–T bonds (T = Si or Al) within the K–A–S–H gel.^33^ A shoulder at approximately 1050–1100 cm^−1^ indicates the presence of unreacted metakaolin. There are a few noticeable differences between the spectra at 3 days and the spectra at 28 days, most prominently the sharp peaks that are seen at 667 cm^−1^ in the SO_4__1 and SO_4__5 samples. It is possible that these peaks are due to the presence of kalistrontite (K_2_Sr(SO_4_)_2_) within the sample, as observed by XRD. The FTIR data appear very similar regardless of the Sr/Al ratio of the samples. This suggests that the K–A–S–H gel structure is not altered by the addition of the SrSO_4_, as observed by FTIR, despite the clear effects on the kinetics observed previously.^18^

#### 3.4.2 X-ray diffraction

The X-ray diffraction data for the geopolymer samples containing SrSO_4_ and cured for 3 and 28 days are shown in Fig. S8, SI. The data show the same features as the XRD data for the geopolymer samples containing Sr(OH)_2_·8H_2_O, with a broad feature between 22° 2*θ* and 35° 2*θ*, arising from diffuse scattering and consistent with the presence of a K–A–S–H gel,^39^ as well as reflections due to anatase (TiO_2_, Powder Diffraction file (PDF) # 01-084-1286) which is present as an impurity in the metakaolin used, and reflections due to strontianite (SrCO_3_, PDF # 00-005-0418). The presence of reflections due to celestine (SrSO_4_, PDF # 04-009-9879) confirms that not all of the SrSO_4_ dissolves into the activating solution. The presence of this becomes greater with increasing Sr/Al ratio, suggesting that the solubility limit is reached between the SO_4__1 and SO_4__2 samples. The presence of arcanite (K_2_SO_4_, PDF #00-024-0703) suggests that the dissolved SO_4_^2−^ anions react with the K^+^ ions in solution to precipitate a solid, crystalline K_2_SO_4_ phase. The presence of this also increases with increasing Sr/Al ratio, suggesting that, as more SrSO_4_ dissolves, there are more SO_4_^2−^ anions available to react with the K^+^ cations as would be expected. This is similar for the kalistrontite (K_2_Sr(SO_4_)_2_, PDF # 04-025-4251), which increases in prominence as mass of SrSO_4_ in the sample increases. The formation of kalistrontite is not unexpected, as it is seen in the literature that deposits of kalistrontite have been found in the presence of SrSO_4_ and K-bearing phases.^59,60^ The presence of this crystalline solid could explain the changes seen to the reaction kinetics observed previously,^18^ as SrSO_4_ is observed to react with the activating solution upon its addition. The crystallisation, and probable resultant precipitation, of this solid, as well as consumption of both K^+^ and Sr^2+^, the two cations within solution that are responsible for the charge balancing of the AlO_4_^−^ sites in the gel phase, may then affect dissolution of metakaolin and formation of the K–A–S–H gel. The presence of reflections due to strontianite (SrCO_3_, PDF # 00-005-0418) in the XRD data is most likely due to the carbonation of the sample *via* contact with air. However, there is very little of this compound present within the sample, providing further evidence that there is a minimal amount of excess ‘free’ Sr within the sample, as the majority of it has either not dissolved (remaining as SrSO_4_), has reacted with the activating solution to form kalistrontite, or has been incorporated into the K–A–S–H gel in place of K. After 28 days, the diffraction patterns show the presence of all of the crystalline phases seen after 3 days. The presence of quartz (SiO_2_, PDF # 01-078-2315) is also seen, an inert compound found in the raw metakaolin precursor.

#### 3.4.3 Solid state nuclear magnetic resonance spectroscopy

##### 3.4.3.1 ^29^Si MAS and ^1^H–^29^Si CP MAS NMR

**Fig. 16:**
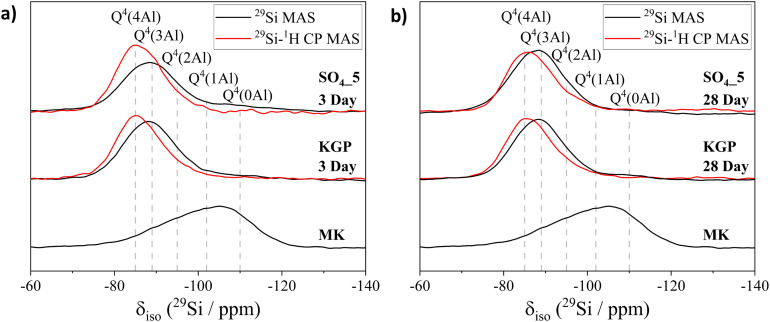
(a) ^1^H–^29^Si CP MAS (shown in red, *B*_0_ = 11.7 T, *ν*_R_ = 12.5 kHz and Hartmann–Hahn contact period *t* = 2.0 ms) and (b) ^29^Si MAS (shown in black, *B*_0_ = 11.7 T, *ν*_R_ = 12.5 kHz) NMR data and associated deconvolutions for geopolymers cured for 3 and 28 days with and without SrSO_4_, and metakaolin.

The ^1^H–^29^Si CP MAS NMR and ^29^Si MAS NMR data for the SO_4__5 and KGP samples cured for 3 and 28 days are shown in Fig. 16. The ^1^H–^29^Si CP MAS NMR data for each geopolymer exhibits a broad resonance centred at *δ*_iso_ = −85.0 ppm and spanning from *δ*_iso_ = −75 to −100 ppm, with a consistent lineshape for all samples. Deconvolution and quantification of the ^1^H–^29^Si CP MAS and ^29^Si MAS NMR data identifies Q^4^(4Al), Q^4^(3Al), Q^4^(2Al), and Q^4^(1Al) sites within an Al-rich (Si/Al ≤ 1.2), fully polymerised K–A–S–H gel,^20,30^ with Si/Al ratios similar to that observed for geopolymers incorporating Sr(OH)_2_·8H_2_O, SrCO_3_, and Sr(NO_3_)_2_. The SO_4__5 sample shows consistently higher Si/Al ratios than KGP at both time points, which may arise from a lower extent of reaction and hence greater proportion of Si sites from unreacted metakaolin in these samples compared to the control samples. This is consistent with the FTIR and XRD data for these samples, as well as previous observations by isothermal calorimetry.^18^ The calculated extent of reaction shows that the metakaolin precursor continues to react between 3 and 28 days curing.

The Si/Al ratio of the SO_4__5 samples after both 3 and 28 days are greater than that of the KGP sample suggesting that the incorporation of SrSO_4_ at such a high Sr/Al ratio slightly decreases the cation binding capability of the gel. This is contrary to what has been seen in previous work, with Walkley *et al.* showing that the Si/Al ratio for K-activated geopolymer systems containing Sr is less than the associated control geopolymer system.^22^ However, this is using much lower concentrations of Sr within the structure, which would have less of an impact on the formation of the K–A–S–H phase.

The SO_4__5 sample appears to have a gel structure dominated by Q^4^(4Al), Q^4^(3Al) and Q^4^(2Al) silicon sites after 3 days but after 28 days, the percentage of Q^4^(2Al) sites drops significantly to a structure dominated by Q^4^(4Al) and Q^4^(3Al) sites. This shows that the Al content of the K–A–S–H gel is increasing over time, agreeing with the Si/Al ratios shown in Table S4, SI. Previous work using zeta potential measurements of dispersions of metakaolin particles in solutions representative of the aqueous phase in the fresh geopolymers studied in this work suggest that the dissolution of the metakaolin is slowed due to the adsorption of Sr^2+^ cations to its surface.^18^ The results here show that this leads to a Si-rich K–A–S–H gel in the early stages of the reaction (due to the presence of excess soluble silica from the activating solution) which becomes more Al-rich as the sample ages. This is consistent with the FTIR and XRD data for these samples, as well as previous observations by isothermal calorimetry.^18^ The calculated extent of reaction shows that the metakaolin precursor continues to react between 3 and 28 days curing.

##### 3.4.3.2 ^27^Al and ^39^K MAS NMR

**Fig. 17:**
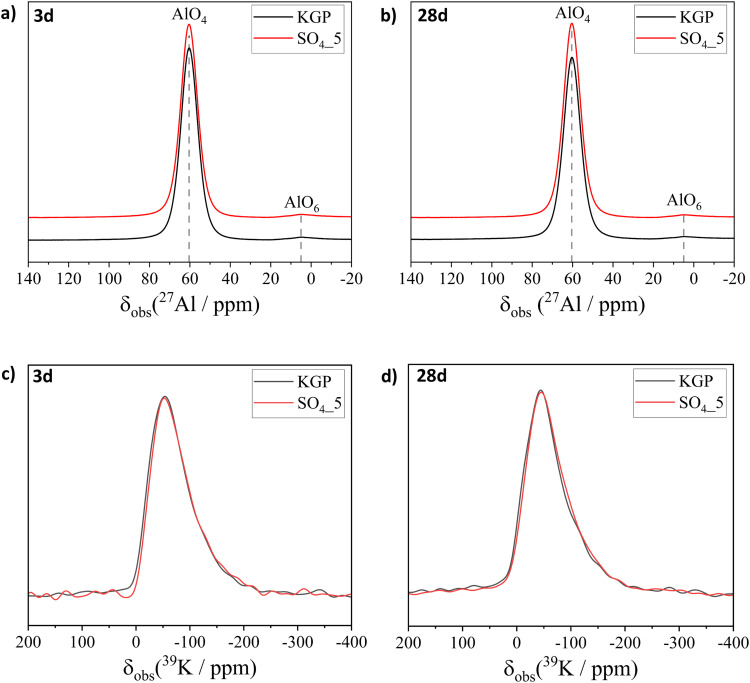
(a) ^27^Al (*B*_0_ = 11.7 T, *ν*_R_ = 12.5 kHz) and (b) ^39^K MAS NMR data (*B*_0_ = 20.0 T, *ν*_R_ = 12.5 kHz) for each geopolymer cured for 3 and 28 days with and without SrSO_4_.

^27^Al and ^39^K MAS NMR data for all samples (Fig. 17) are nearly identical to those obtained for geopolymers samples incorporating Sr(OH)_2_·8H_2_O, indicating that Al exists within tetrahedral (q^4^) sites in the K–A–S–H type gel as well as octahedral Al sites within remnant unreacted metakaolin, and K exists in charge-balancing extra-framework sites within the K–A–S–H-type gel.^19,44^ This is supported by the findings of both the ^29^Si MAS and ^1^H–^29^Si CP MAS NMR data for these samples. No differences in either resonance are observed when comparing samples with and without Sr.

**Fig. 18:**
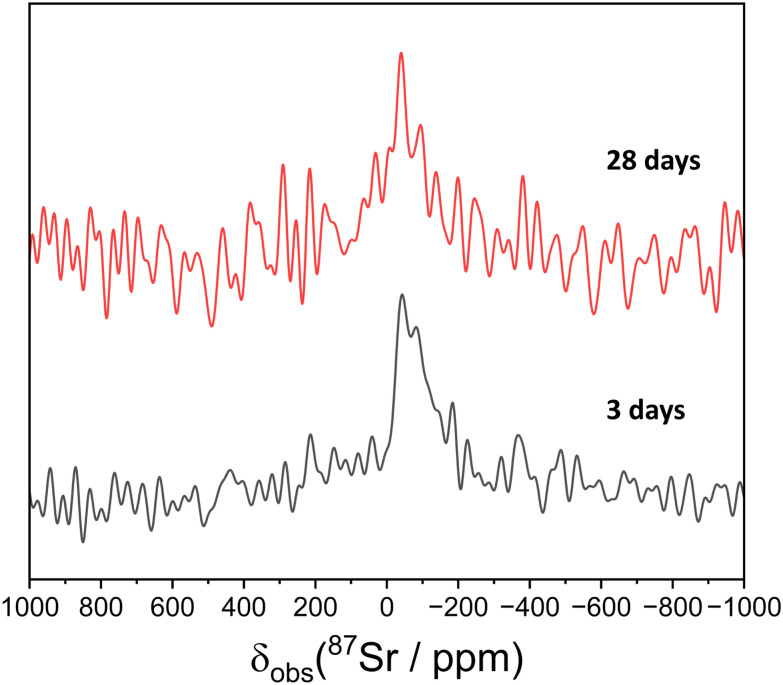
^87^Sr MAS NMR data (*B*_0_ = 20.0 T, *ν*_R_ = 12.5 kHz with 1024 data points transformed (out of 8192 data points obtained) and line broadening of 100 Hz applied) for the SO_4__5 geopolymer sample cured for 3 and 28 days.

The ^87^Sr NMR data for the SO_4__5 sample after curing for 3 and 28 days are shown in Fig. 18. As with the data for geopolymers incorporating Sr(OH)_2_·8H_2_O and Sr(NO_3_)_2_, the ^87^Sr MAS NMR data for the SO_4__5 sample after curing for 3 and 28 days exhibits a single distinct resonance with a broad quadrupolar lineshape, centred at approximately *δ*_obs_ = −33 ppm. This is broadly consistent with the chemical shift and FWHM observed for ^87^Sr MAS NMR data for SrSO_4_,^61^ suggesting that this phase may be responsible for the resonance observed in the data here. The ^87^Sr MAS NMR data shown in Fig. 13 exhibit significant intensity at *δ*_obs_ = 0 ppm, as a shoulder on the main resonance, consistent with that of the SrSiO_3_ phase observed in nuclear waste glasses.^54^ However, similar to observations for the OH_5 and NO_3__5 samples, this shoulder on the data shown here for the SO_4__5 sample is not significantly above the spectral noise, and so no firm conclusions can be drawn from this data about the existence of this site.

#### 3.4.4 X-ray absorption near-edge structure (XANES) spectroscopy

**Fig. 19:**
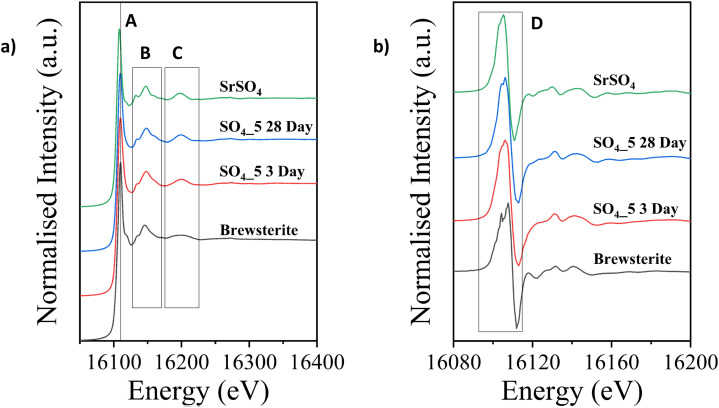
(a) Normalised X-ray absorption spectra for the geopolymer samples loaded with SrSO_4_, reagent grade SrSO_4_, and the mineral brewsterite-Sr, and (b) the 1^st^ derivative of these spectra.

The normalised spectra for the Sr K-edge XAS analysis of the SO_4__5 sample after both 3 and 28 days of reaction compared to both the SrSO_4_ reagent and brewsterite-Sr ((Sr,Ba)_2_Al_4_Si_12_O_32_·10H_2_O) are shown in Fig. 19.^53^ The Sr K-edge here displays an absorption edge that rises smoothly to a maximum featuring a singular peak. When initially focusing on the peak maximum in Fig. 19a, labelled as A, the value of the peak appears to move to slightly higher energies when the SrSO_4_ is included in the geopolymer mix when compared to the SrSO_4_ reagent. The energy at which the brewsterite-Sr mineral reaches its maximum peak is comparable to the SO_4__5 samples. This peak can be explored in more detail in Fig. 19b, which displays the 1^st^ derivative of this peak. This allows us to further distinguish the features of the leading edge, and shows that there is a potential feature here, most notably in the brewsterite standard. However, the line shape of the spectra for the SO_4__5 samples both follow that of the SrSO_4_ more closely, with the doublet being far closer to the tip of the peak in both samples.

When exploring the regions above the absorption edge, inspection of the region labelled B in Fig. 19a does not provide a great deal of insight due to the similarities presented here between the SrSO_4_ standard and the brewsterite standard. However, region C shows that the SO_4__5 samples at both time points relate more closely to the SrSO_4_ standard than the brewsterite. The spectra here suggest that the Sr within the SO_4__5 samples are likely to exist primarily in SrSO_4_, as well as incorporated into the K–A–S–H gel as charge balancing cations within a brewsterite-type structure.

#### 3.4.5 Electron probe microanalysis

**Fig. 20:**
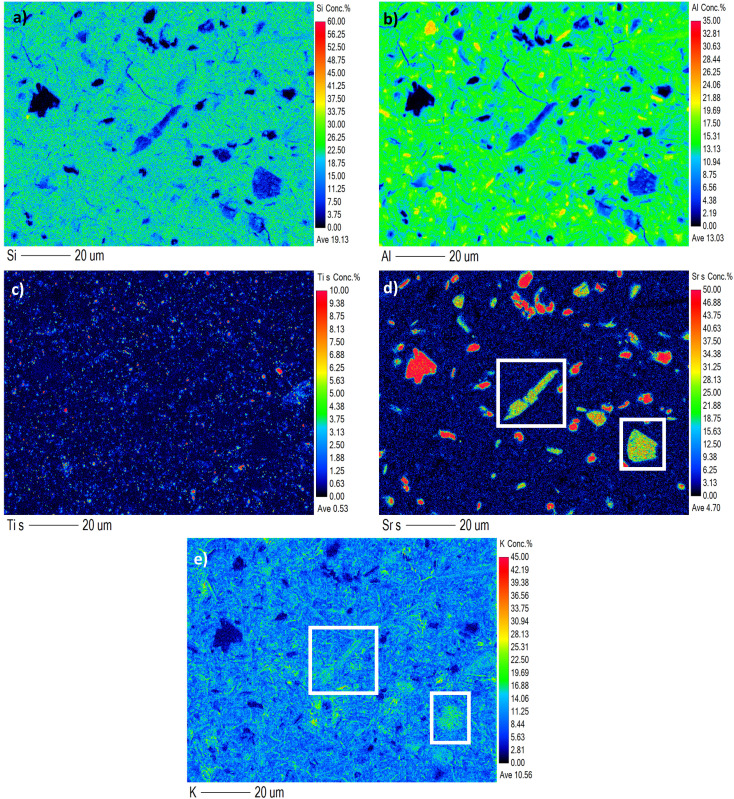
(a) Electron probe microanalysis images generated for the SO_4__5 geopolymer cured for 28 days showing distribution of (a) aluminium, (b) silicon, (c) titanium, (d) strontium, and (e) potassium within the sample.

EPMA images of the SO_4__5 sample cured for 28 days are shown in Fig. 20, and the data exhibit a distribution of Al, Si, and K consistent with the amorphous K–A–S–H gel framework, with unreacted metakaolin particles (observed as Al-rich regions), similar to the data for geopolymers incorporating Sr(OH)_2_·8H_2_O, SrCO_3_, and Sr(NO_3_)_2_. Sr is present within the sample in multiple phases. Firstly, the regions of very high Sr content, depicted by the red regions in the image, suggest the presence of unreacted and undissolved SrSO_4_ salt which was added as the reagent. It is likely that this phase is SrSO_4_ due to the low concentration of the other elements in these areas. This phase is seen throughout the sample, and suggests that the SrSO_4_ mixed within the geopolymer gel, but has become encapsulated within the gel as opposed to becoming incorporated chemically into the structure in place of the K^+^ cations as the charge balancing ion. To add to this, the widespread concentration of K shown in image (e) suggests that it is acting as the charge balancing cation in the K–A–S–H gel in the majority of cases. An additional phase is observed that contains relatively high concentrations of both Sr and K (indicated by the white boxes in both images). This phase is potentially that of the kalistrontite that is seen in the XRD analysis, and this is supported by the small concentration of Si that can be seen in these regions. This finding provides further evidence for the reaction between the SrSO_4_ salt and the activating solution, the only reagent containing K, which is likely to be causing the changes in reaction kinetics observed previously.^18^

## 4 Conclusions

This study provides a comprehensive analysis of the impact of Sr salt chemistry on the development, mechanisms of reaction, and nanostructural development during K–A–S–H gel formation, of potassium silicate-activated geopolymers. Across all samples, the primary binding phase is a structurally disordered, highly cross-linked K–A–S–H gel. The gel consists of a fully polymerised aluminosilicate framework where Al exists in tetrahedral coordination and Si is distributed across various Q^4^(*m*Al) environments.

The mechanism of Sr incorporation is highly dependent on the solubility of the initial salt. Soluble salts (strontium nitrate and strontium hydroxide) release Sr^2+^ ions that can be chemically incorporated into the geopolymer framework as charge-balancing cations, forming pseudo-zeolitic (brewsterite-type) local structures. Less soluble salts (carbonate and sulfate) remain largely unreacted and become physically encapsulated within the geopolymer matrix. The presence of Sr(NO_3_)_2_ and SrSO_4_ can alter the early-stage formation of the K–A–S–H gel by delaying metakaolin dissolution by forming a physical or electrostatic shielding layer on the precursor surface, leading to an initially Si-rich gel that becomes increasingly Al-rich as reaction progresses.

In specific cases, chemical reactions between the Sr salts and the alkaline activator lead to new crystalline phases, such as kalistrontite (K_2_Sr(SO_4_)_2_) in sulfate-bearing samples or niter (KNO_3_) in nitrate-bearing samples. The ability of K–A–S–H geopolymers to accommodate Sr through both chemical substitution in the gel framework and physical encapsulation of discrete salt particles highlights their potential as robust materials for the long-term immobilisation of ^90^Sr-bearing radioactive waste streams.

## Conflicts of interest

There are no conflicts to declare.

## Supplementary Material

DT-055-D6DT00775A-s001

## Data Availability

All data supporting this article have been included as part of the main manuscript. Supplementary information (SI) is available. See DOI: https://doi.org/10.1039/d6dt00775a.
