# Supplementary material for: High-field multinuclear MAS NMR and synchrotron XANES reveal the influence of strontium salt chemistry on geopolymer nanostructure
Source: Dalton Trans. 2026 Jun 17;55(28):10673–95. doi: 10.1039/d6dt00775a (PMC13330456; doi:10.1039/d6dt00775a)
Supplement: DT-055-D6DT00775A-s001 [file DT-055-D6DT00775A-s001.pdf]

Supplementary Information (SI) for Dalton Transactions.
This journal is © The Royal Society of Chemistry 2026

Supporting Information for

High-field multinuclear MAS NMR and synchrotron XANES reveal the influence of strontium salt chemistry on geopolymer nanostructure

Kyle T. O'Donoghue[1], Daniel A. Geddes[1], Tom J. Wilkinson[1], Martin C. Stennett[1], Byoungkwan Kim[1], Dinu Iuga[2], Martin Hayes[3], Brant Walkley[1]*

[1]*School of Chemical, Materials and Biological Engineering, The University of Sheffield, Sheffield, UK*

[2]*Department of Physics, University of Warwick, Coventry, UK*

[3]*United Kingdom National Nuclear Laboratory, Warrington, UK*

Corresponding author: Email: b.walkley@sheffield.ac.uk

1. Effect of addition of strontium hydroxide octahydrate to geopolymers

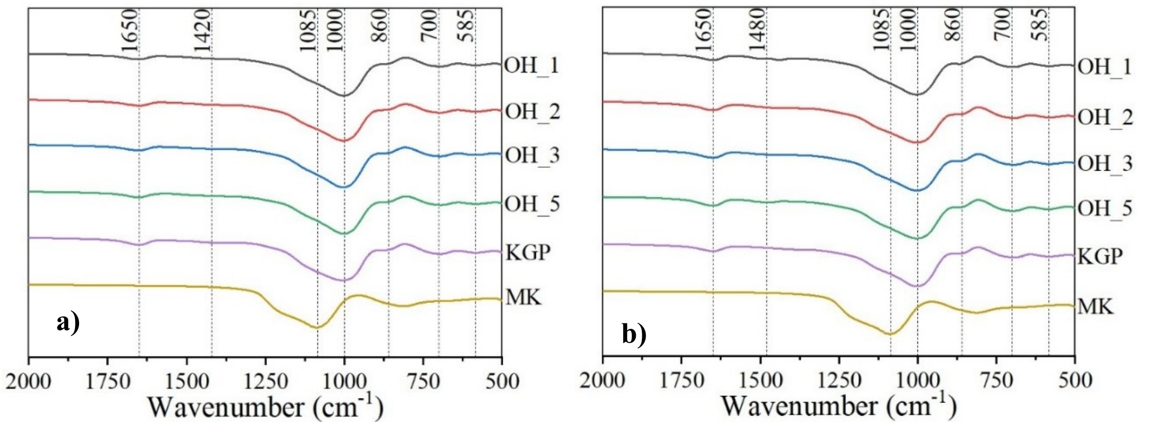

Figure S1: FTIR data for the geopolymers cured for a) 3 and b) 28 days, as a function of the amount of $Sr(OH)_2 \cdot 8H_2O$ (wt. %) in the formulation, and unreacted metakaolin (MK).

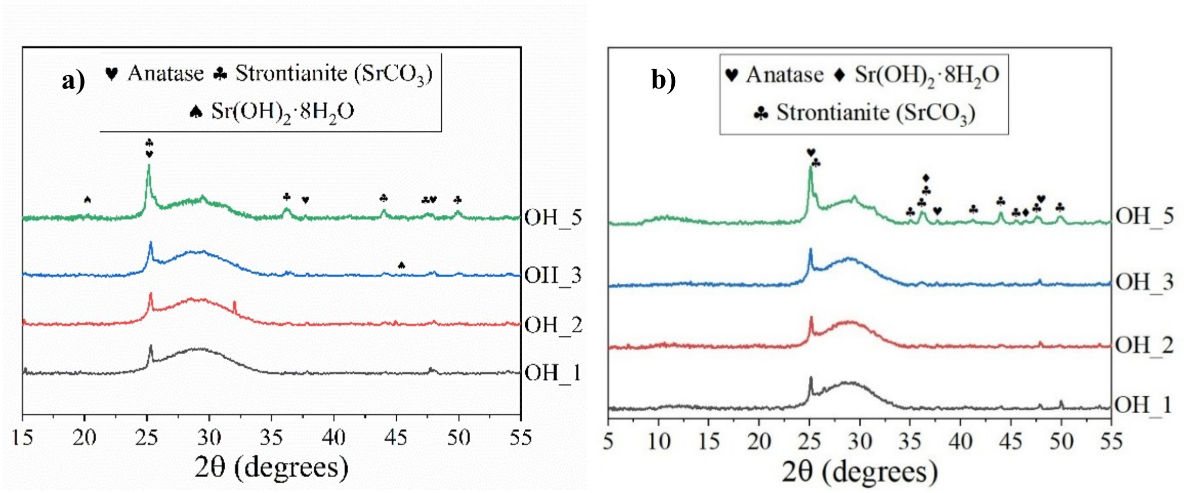

Figure S2: X-ray diffraction data for the geopolymers cured for a) 3 and b) 28 days, as a function of the amount of $Sr(OH)_2 \cdot 8H_2O$ (wt. %) in the formulation.

Table S1: Relative integral areas for Q^4(mAl) sites and Si/Al ratios within K-A-S-H in each sample, as well as extent of reaction (mol. %), calculated from the deconvoluted ^{29}Si MAS NMR spectra

		Relative Integral Area (%)*					
**Sample**	**Time point**	**Q^4(4Al**	**Q^4(3Al)**	**Q^4(2Al)**	**Q^4(1Al)**	**Si/Al**	**Extent of**

		)					reaction (%)
KGP	3 Days	25	54	20	1	**1.32**	**82.17**
OH_5	3 Days	27	35	34	4	**1.40**	**87.28**
KGP	28 Days	28	44	26	2	**1.34**	**88.00**
OH_5	28 Days	31	32	30	7	**1.39**	**87.44**

* Estimated error due to spectral noise is ± 2 %

2. Effect of addition of strontium carbonate to geopolymers

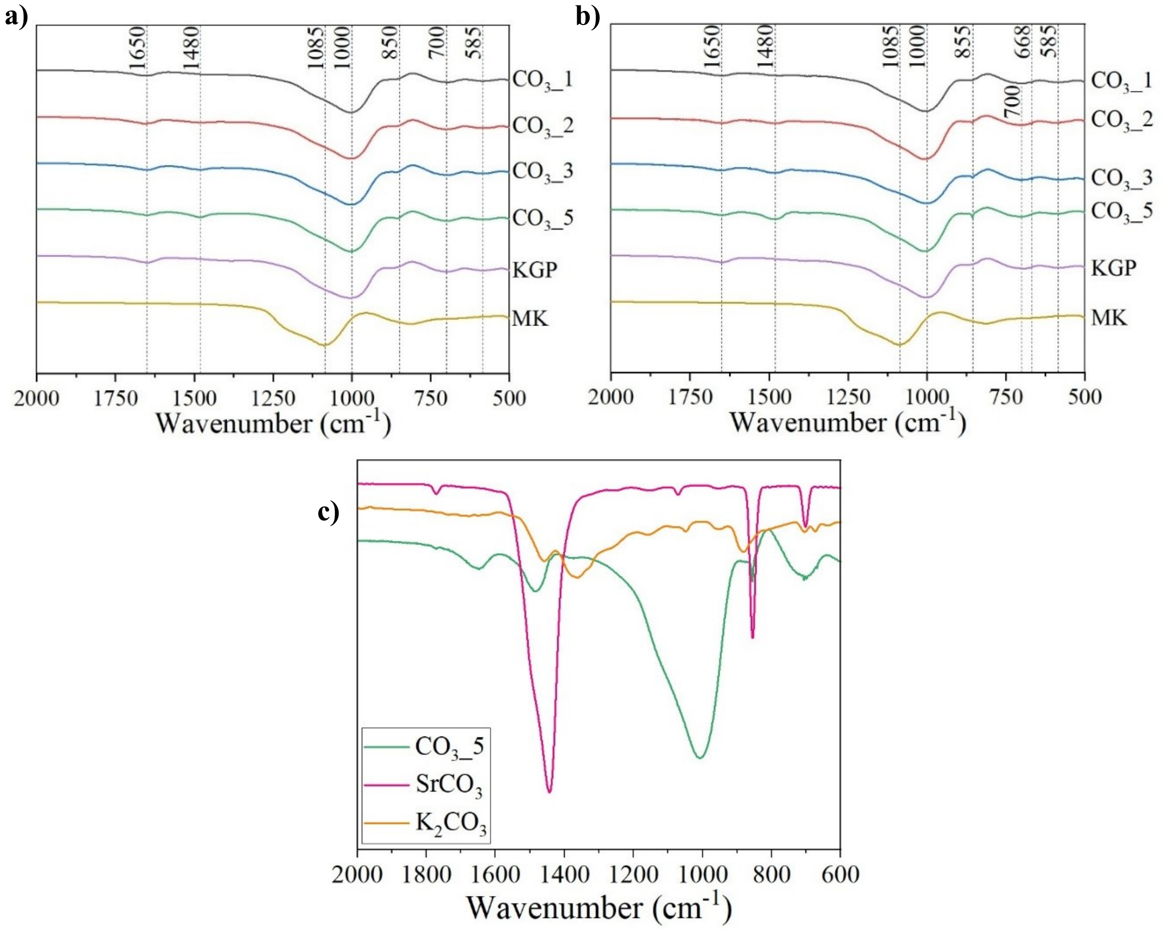

Figure S3: FTIR data for the geopolymers cured for a) 3 and b) 28 days, as a function of the amount of $SrCO_3$ (wt. %) in the formulation, and unreacted metakaolin (MK), as well as c) FTIR data for the CO_3_5 sample compared against that of reagents $SrCO_3$ and K_2CO_3.

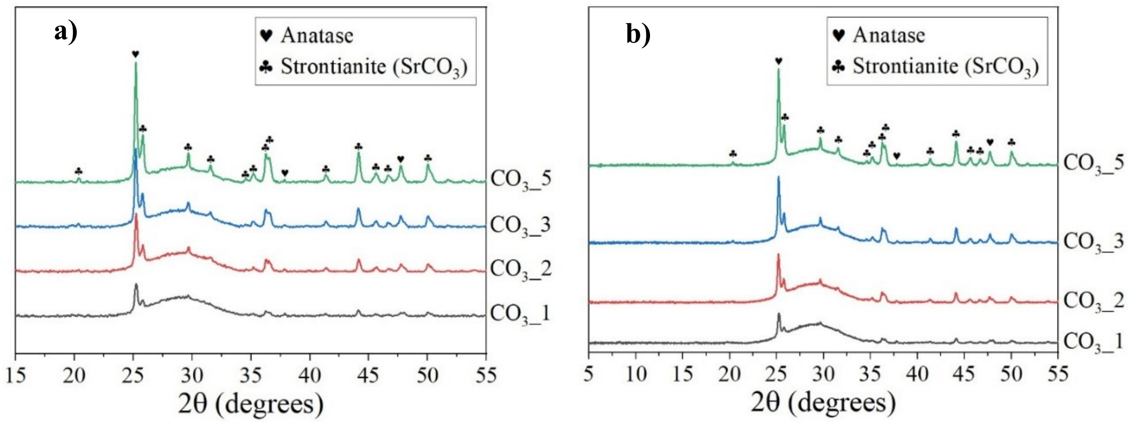

Figure S4: X-ray diffraction data for the geopolymers cured for a) 3 and b) 28 days, as a function of the amount of $SrCO_3$ (wt. %) in the formulation.

Table S2: Relative integral areas for Q^4(mAl) sites and Si/Al ratios within K-A-S-H in each sample, as well as extent of reaction (mol. %), calculated from the deconvoluted ^{29}Si MAS NMR spectra

		Relative Integral Area (%)					
**Sample**	**Time point**	**Q^4(4Al)**	**Q^4(3Al)**	**Q^4(2Al)**	**Q^4(1Al)**	**Si/Al**	**Extent of reaction (%)**
KGP	3 Days	25	54	20	1	**1.32**	**82.17**
CO_3_5	3 Days	24	49	23	4	**1.37**	**80.73**
KGP	28 Days	28	44	26	2	**1.34**	**88.00**
CO_3_5	28 Days	24	48	27	1	**1.36**	**83.01**

3. Effect of addition of strontium nitrate to geopolymers

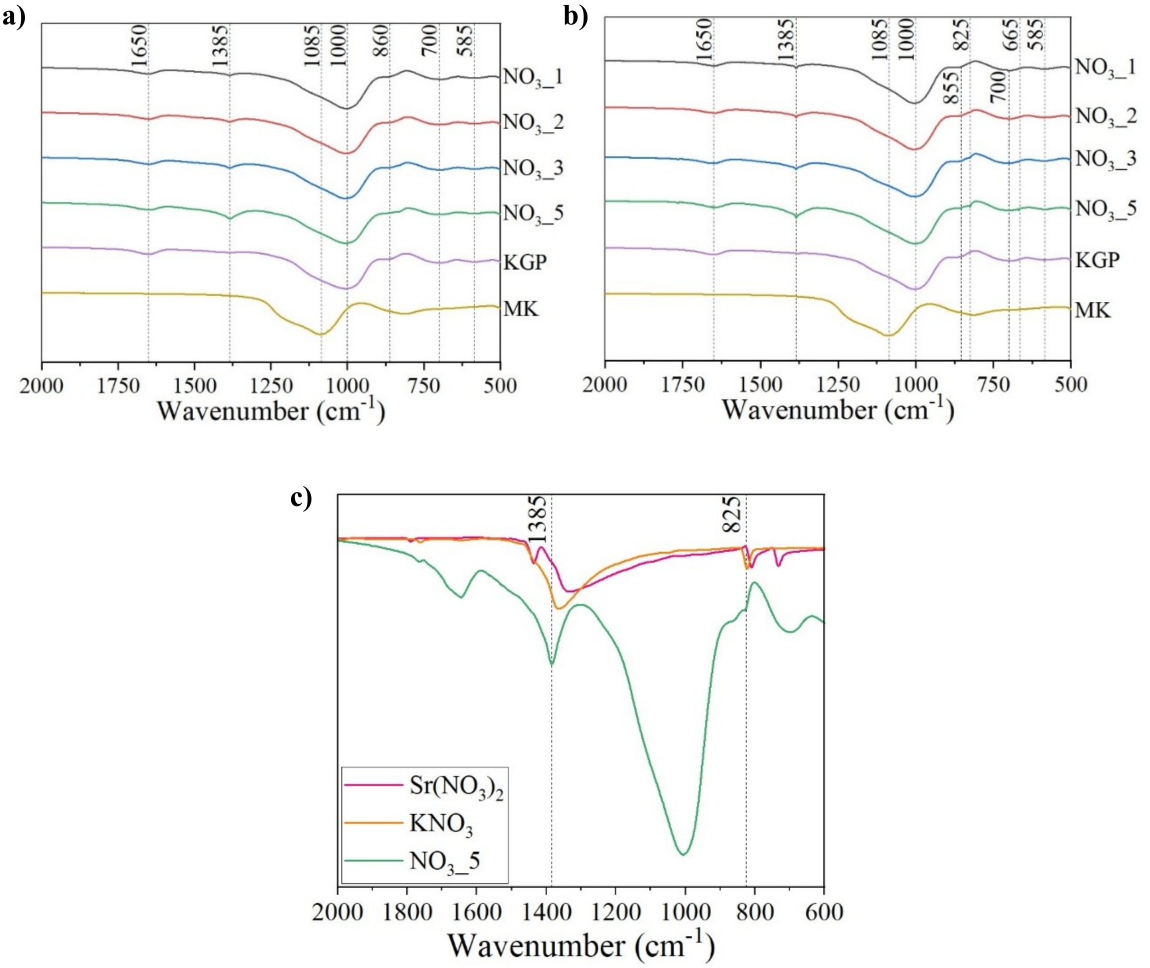

Figure S5: FTIR data for the geopolymers cured for a) 3 and b) 28 days, as a function of the amount of $Sr(NO_3)_2$ (wt. %) in the formulation, and unreacted metakaolin (MK). FTIR data for the reagents used to produce the geopolymer samples are shown in c).

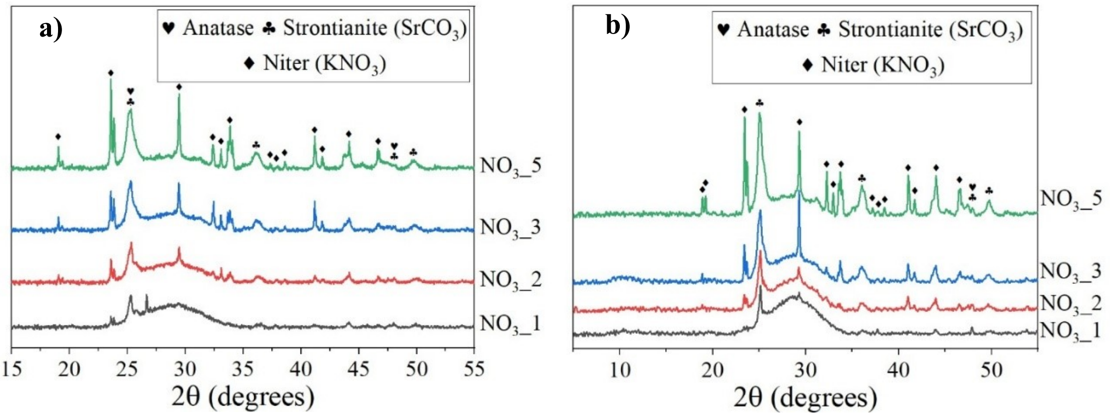

Figure S6: X-ray diffraction data for the geopolymers cured for a) 3 and b) 28 days, as a function of the amount of $Sr(NO_3)_2$ (wt. %) in the formulation.

Table S3: Relative integral areas for Q^4(mAl) sites and Si/Al ratios within K-A-S-H in each sample, as well as extent of reaction (mol. %), calculated from the deconvoluted ^{29}Si MAS NMR spectra

		Relative Integral Area (%)					
**Sample**	**Time point**	**Q^4(4Al)**	**Q^4(3Al)**	**Q^4(2Al)**	**Q^4(1Al)**	**Si/Al**	**Extent of reaction (%)**
KGP	3 Days	25	54	20	1	**1.32**	**82.17**
NO_3_5	3 Days	30	38	30	2	**1.35**	**73.32**
KGP	28 Days	28	44	26	2	**1.34**	**88.00**
NO_3_5	28 Days	39	40	20	1	**1.26**	**77.73**

4. Effect of addition of strontium sulfate to geopolymers

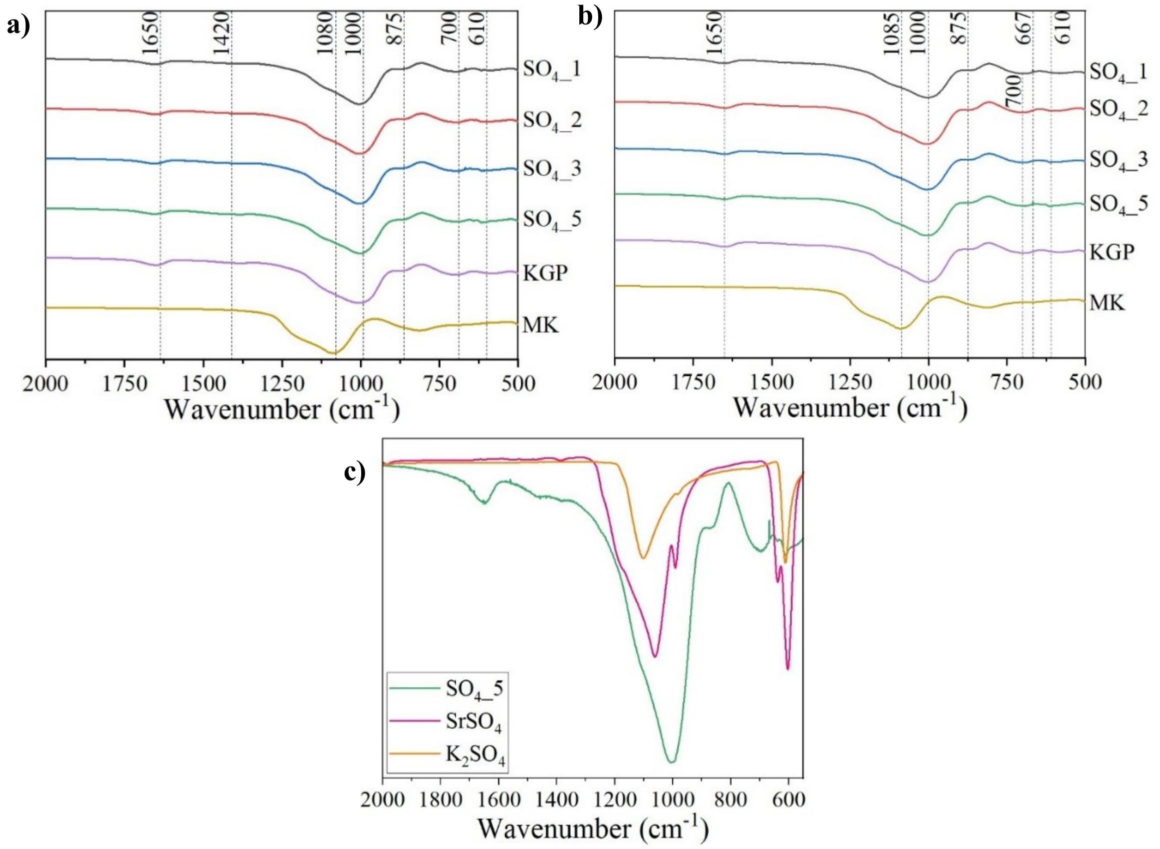

Figure S7: FTIR data for the geopolymers cured for 3 and 28 days, as a function of the amount of $SrSO_4$ (wt. %) in the formulation, the control geopolymer sample, unreacted metakaolin (MK), and $SrSO_4$ and K_2SO_4 reagents.

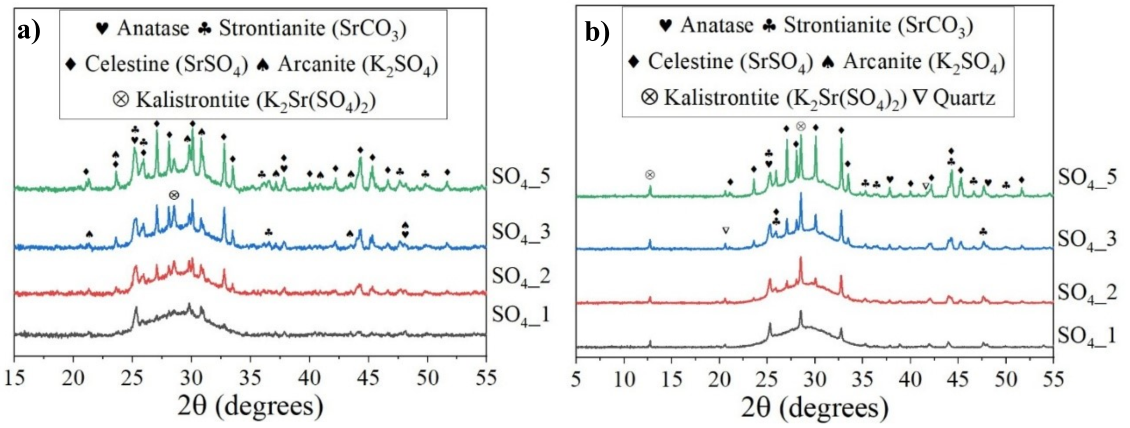

Figure S8: X-ray diffraction data for the geopolymers cured for a) 3 and b) 28 days, as a function of the amount of $SrSO_4$ (wt. %) in the formulation.

Table S4: Relative integral areas for Q^4(mAl) sites and Si/Al ratios within K-A-S-H in each sample, as well as extent of reaction (mol. %), calculated from the deconvoluted ^{29}Si MAS NMR spectra

		Relative Integral Area (%)					
**Sample**	**Time point**	**Q^4(4Al)**	**Q^4(3Al)**	**Q^4(2Al)**	**Q^4(1Al)**	**Si/Al**	**Extent of reaction (%)**
KGP	3 Days	25	54	20	1	**1.32**	**82.17**
SO_4_5	3 Days	25	33	37	5	**1.37**	**84.40**
KGP	28 Days	28	44	26	2	**1.34**	**88.00**
SO_4_5	28 Days	31	34	28	7	**1.36**	**86.61**
